# The mitophagy pathway and its implications in human diseases

**DOI:** 10.1038/s41392-023-01503-7

**Published:** 2023-08-16

**Authors:** Shouliang Wang, Haijiao Long, Lianjie Hou, Baorong Feng, Zihong Ma, Ying Wu, Yu Zeng, Jiahao Cai, Da-wei Zhang, Guojun Zhao

**Affiliations:** 1 The Sixth Affiliated Hospital of Guangzhou Medical University, Qingyuan City People’s Hospital, Qingyuan, Guangdong China; 2 Xiangya Hospital, Central South University, Changsha, Hunan China; 3 Group on the Molecular and Cell Biology of Lipids and Department of Pediatrics, Faculty of Medicine and Dentistry, University of Alberta, Edmonton, Alberta Canada

**Keywords:** Molecular medicine, Cell biology

## Abstract

Mitochondria are dynamic organelles with multiple functions. They participate in necrotic cell death and programmed apoptotic, and are crucial for cell metabolism and survival. Mitophagy serves as a cytoprotective mechanism to remove superfluous or dysfunctional mitochondria and maintain mitochondrial fine-tuning numbers to balance intracellular homeostasis. Growing evidences show that mitophagy, as an acute tissue stress response, plays an important role in maintaining the health of the mitochondrial network. Since the timely removal of abnormal mitochondria is essential for cell survival, cells have evolved a variety of mitophagy pathways to ensure that mitophagy can be activated in time under various environments. A better understanding of the mechanism of mitophagy in various diseases is crucial for the treatment of diseases and therapeutic target design. In this review, we summarize the molecular mechanisms of mitophagy-mediated mitochondrial elimination, how mitophagy maintains mitochondrial homeostasis at the system levels and organ, and what alterations in mitophagy are related to the development of diseases, including neurological, cardiovascular, pulmonary, hepatic, renal disease, etc., in recent advances. Finally, we summarize the potential clinical applications and outline the conditions for mitophagy regulators to enter clinical trials. Research advances in signaling transduction of mitophagy will have an important role in developing new therapeutic strategies for precision medicine.

## Introduction

Macroautophagy, as an evolutionarily conserved pathway, involves the process of lysosomal degradation and cellular component recycling.^1^ Mitophagy is a kind of macroautophagy, which selectively transports mitochondria to lysosomes for degradation.^2^ Mitophagy is activated when mitochondrial damage exceeds the capabilities of other quantity and quality control methods, or when mitochondria are removed for cellular metabolic purposes.^3^ Different from general non-selective autophagy, mitophagy requires the gathering of specific receptor proteins on the surface of mitochondria and the activation of specific signaling pathways.^3^

Mitophagy-mediated mitochondrial elimination plays a significant part in numerous processes, such as inflammation, metabolic transitions, and cellular reprogramming.^4^ Damaged mitochondria not only lack the ability to produce ATP and other biosynthetic products, but also release higher levels of reactive oxygen species (ROS).^5^ If ROS cannot be scavenged in time and accumulates in cells, it will result in apoptosis.^5^ Mitophagy maintains mitochondria in an optimal condition by removing dysfunctional or excessive mitochondria. The homeostasis of mitochondria is sustained by an equilibrium of removal and bioproduction, which can be disrupted by uncontrolled mitophagy.^2,4^ This brings about mitochondrial suboptimal state, leading to diseases of the nervous system, cardiovascular (or heart), lung, liver, kidney, skeletal muscle, etc.^4^ The removal of mitochondria in a timely and accurate manner is critical for cell survival in response to changes in developmental, bioenergetic, and environmental conditions. Therefore, cells have evolved diverse pathways to ensure the prompt and precise activation of mitophagy in response to various stimuli.^3^

Recently, there has been a better understanding of how mitophagy is regulated. Preliminary research progress has been made in the mechanistic link between mitophagy and disease and the application of mitochondrial regulators to treat diseases in animal models.^3,4,6^ However, few articles summarize how alterations in mitophagy affect disease development, and interventions specifically targeting the regulation of mitophagy are unavailable in clinical trials. Here, we summarize the signaling pathways and mechanisms that regulate mitophagy. Furthermore, we discuss how alterations in mitophagy affect mitochondrial homeostasis and disease development. Finally, we discuss the necessary conditions and unanswered questions for mitophagy regulators that can potentially enter clinical trials in the future.

## A brief history and milestones of mitophagy

**Fig. 1:**
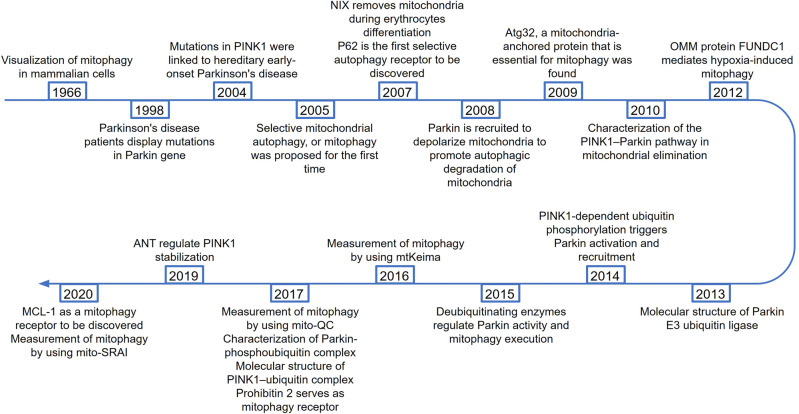
The timeline of some seminal contributions in mitophagy-related research

Christian de Duve named the autophagosome transmission of cellular components “autophagy” in 1966.^7^ There are three primary forms of autophagy: microautophagy, chaperone-mediated autophagy, and macroautophagy.^1^ Macroautophagy, generally known as autophagy, is the most common form of autophagy. Initially, macroautophagy (hereinafter referred to as “autophagy”) was considered to be a nonselective process of bulk degradation. Under particular circumstances, autophagy of protein aggregates and even organelles were seen, suggesting that degradation might be selective instead of always stochastic.^4^ Xue et al. found that mitochondria were selectively cleared in neurons and HeLa cells following caspase inhibitor treatment, independent of the stimulus that triggered apoptosis.^8^ Elmore et al. reported that when there is mitochondrial damage, the opening of the mitochondrial permeability transition pore and early depolarization were induced, causing mitochondrial selective engulfment via autophagosomes in hepatocytes.^9^ These two studies indicate that damaged mitochondrial are likely to be cleared by a specific pathway different from general autophagy. Lemasters first proposed mitophagy in 2005, and pointed out that mitochondrial damage is the signal to initiate mitophagy.^10^ Parkinson protein 2 (Parkin) was recruited to mitochondrial depolarization when induced by mitochondrial damage to promote autophagic degradation of mitochondria.^11^ The first mitophagy receptor, autophagy-related (ATG) protein ATG32, was discovered in yeast.^12^ Since then, massive mitophagy receptors have been discovered, including BNIP3-like (NIX, known as BNIP3L as well),^13^ FUN14 domain contains 1 (FUNDC1),^14^ Prohibitin 2 (PHB2)^15^ and Myeloid cell leukemia-1 (MCL-1),^16^ etc. (Fig. 1).

## Pathway and mechanism of mitophagy

In 2008, Youle et al. showed that Parkin was recruited to depolarize mitochondria to promote autophagic degradation of mitochondria.^11^ This is considered a landmark study in mitophagy. From then on, research on mitophagy has kept on developing, and numerous mitophagy pathways have been discovered.

### PINK1-Parkin-mediated mitophagy

**Fig. 2:**
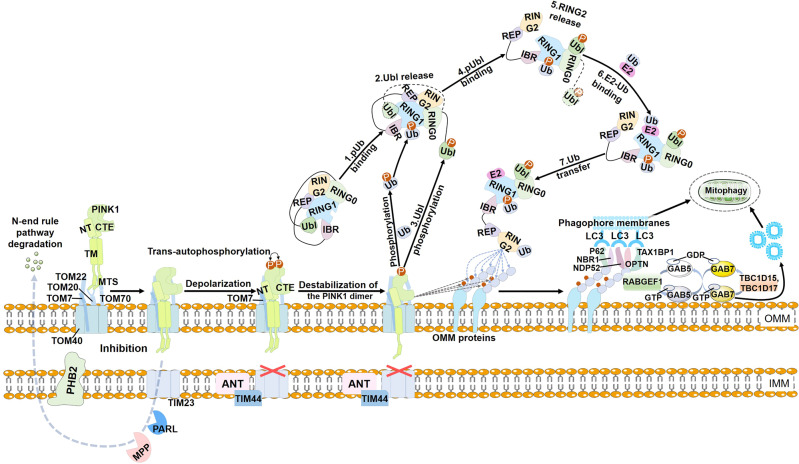
PINK1-Parkin‐mediated mitophagy. With the help of TOM22 and TOM70, TOM20 recognizes the MTS sequence and guides PINK1 into the translocation pore formed by TOM40, which transfers PINK1 to the TIM23 complex in the IMM. Then, PINK1 is sequentially cleaved by the MPP and the PARL, followed by the N-terminal regular degradation pathway. PHB2 binds to PARL to prevent it from directly processing PINK1 in the IMM. When mitochondria are damaged (e.g., depolarization of the mitochondrial membrane), the ANT complex inhibits the translocation of PINK1 to TIM23 via interaction with TIM44. Meanwhile, CTE interaction in PINK1 binds to TOM7, resulting in the stabilization of PINK1 on the OMM. Then, PINK1 undergoes trans-autophosphorylation. Monomeric PINK1 phosphorylates Ub, and then pUb binds RING1 to release Ubl. The Ubl is phosphorylated by PINK1, resulting in the release of the RING2 and exposure of the E2 interaction surface in the RING1. The RING2 then receives ubiquitin from E2 via a thioester linkage and transfers it to the substrate. Parkin is activated to ubiquitinate a number of OMM proteins. These ubiquitin proteins are further phosphorylated by PINK1, which recruits more Parkin to mitochondria and thus generates more ubiquitin chains. Finally, ubiquitin chains on mitochondria are recognized by autophagic adapters (P62, NBR1, NDP52/CALCOCO2, TAX1BP1, and OPTN), leading to mitophagy. RABGEF1 can be recruited to damaged mitochondria via binding to the downstream of Parkin by ubiquitination, which then directs the downstream Rab5 and Rab7 to the damaged mitochondria. Recruited Rab7 promotes Atg9-mediated vesicle assembly and LC3-labeled autophagy membrane encapsulation

Serine/threonine PTEN-induced putative kinase 1 (PINK1) is a kinase encoded by the PARK6 gene, and Parkin is an E3 ubiquitin ligase encoded by the PARK2 gene.^17^ Mutations in PINK1 and Parkin are the earliest genetic events associated with autosomal recessive early-onset Parkinson’s disease.^17,18^ Studies on gene destruction in Drosophila have shown that Parkin performs as a downstream component of PINK1 in mitophagy signaling.^19–21^ Since then, numerous studies have focused on how PINK1 directly regulates Parkin. It is essential for mitophagy in the PINK1-Parkin regulatory pathway that ubiquitin chains are assembled on mitochondria. This assembly contains three crucial components, PINK1 as a mitochondrial damage sensor, Parkin as a signal amplifier, and ubiquitin chains as a signal effector. Together, they determine how damaged mitochondria activate mitophagy.^22^ In addition, deubiquitinating enzymes (DUBs) regulate mitophagy by deubiquitinating Parkin or its targets on the mitochondrial, and Parkin can mediate nuclear translocation of transcript factor EB (TFEB), thereby upregulating the expression of genes associated with lysosome biogenesis (Fig. 2).^23,24^

#### PINK1 as a mitochondrial damage sensor

PINK1 includes an N-terminal mitochondrial targeting sequence (MTS). Under the help of translocases of outer mitochondrial membrane 22 (TOM22) and TOM70, TOM20 identifies the MTS sequence and directs PINK1 into the translocation pore formed by TOM40, which removes PINK1 to the TIM23 complex in the inner mitochondrial membrane (IMM).^25,26^ After that, PINK1 is sequentially cleaved by the mitochondrial processing peptidase (MPP) and the presenilin-associated rhomboid-like (PARL), and then degraded through the N-end rule pathway.^27,28^ When mitochondria are damaged (e.g., depolarization of the mitochondrial membrane), the adenine nucleotide translocator (ANT) complex inhibits the translocation of PINK1 to TIM23 by interacting with TIM44, a well-known regulator of TIM23 polypeptide import.^29^ Meanwhile, the C-terminal extension interaction in PINK1 binds to TOM7, stabilizing PINK1 which is on the outer mitochondrial membrane (OMM).^30^ With the help of the TOM complex, PINK1 undergoes trans-autophosphorylation (Ser228 in the human protein), which triggers a conformational change in the N-lobe and subsequently destabilizes the PINK1 dimer. As a result, phosphorylated PINK1, acting as a Parkin kinase and monomeric ubiquitin kinase, initiates mitophagy (Fig. 2).^31^

#### Parkin as a signal amplifier

Parkin consists of ubiquitin‐like (Ubl), repressor element of Parkin (REP), in‐between‐RING (IBR), really interesting new gene 0 (RING0), RING1, and RING2 domains.^6^ In healthy mitochondria, Parkin is diffusely distributed in the cytosol as an autoinhibited form. When interacting with phosphorylated ubiquitin, Parkin undergoes intramolecular structural remodeling to release Ubl, which is readily phosphorylated by PINK1 at Ser65, resulting in the release of RING2 and exposure of the E2 interaction surface in RING1.^32^ RING2 then receives ubiquitin from E2 via a thioester linkage and subsequently transfers it to substrates.^33^ Through this cascade of structural remodeling, Parkin transforms from a self‐inhibiting dormant enzyme to an active E3 to ubiquitinate a number of OMM proteins, such as mitofusins1 (MFN1),^34^ mitofusins2 (MFN2),^34^ mitochondrial Rho-GTPase 1 (Miro1),^35^ and voltage-dependent anion channel 1 (VDAC1).^36^ These ubiquitinated proteins are further phosphorylated by PINK1 to recruit more Parkin to mitochondria, thereby generating more ubiquitin chains.^22^ Notably, Parkin can ubiquitinate artificial mitochondria‐targeted exogenous proteins green fluorescent protein (GFP) and myelin basic protein (MBP), indicating that the E3 ligase does not require a consensus substrate recognition sequence.^37^ In contrast, Parkin is spatially selective for depolarized mitochondria. This unique selectivity appears to be the key to Parkin’s efficient and rapid ubiquitylation of dysfunctional mitochondria.

#### Ubiquitin chains as a signal effector

Autophagosomes encapsulate ubiquitin-tagged mitochondria for lysosomal degradation. However, the ubiquitin chains themselves do not bind to isolated autophagic membrane or ATG8 family proteins conjugated to the isolated membrane. Thus, ubiquitinated cargoes must be tethered to the autophagic membrane via some molecular mechanisms.^38^ Autophagy adapters are defined as proteins possessing both a ubiquitin-binding domain (UBD) that recognizes ubiquitin-tagged mitochondria and an LC3 interacting region (LIR) that interacts with ATG8 family proteins, which mainly includes sequestosome 1 (P62/SQSTM1),^39^ neighbor of BRCA1 gene 1 (NBR1),^40^ nuclear dot protein 52 (NDP52/CALCOCO2),^41^ human T-cell leukemia virus type I binding protein 1 (TAX1BP1),^42^ and optineurin (OPTN).^43^

P62 was the first identified selective autophagy receptor. Accumulation of P62-enriched ubiquitin-positive aggregates was found in cells with defective autophagy due to ATG7 deficiency, and genetic ablation of P62 reduced ubiquitin-positive aggregates.^44,45^ P62 is primarily degraded in lysosomes via autophagy, indicating that it functions as an autophagy adapter to transport ubiquitin-positive inclusions to lysosomes. Aside from the UBD and LIR domains, P62 contains an N-terminal PB1 domain, which can undergo homo-oligomerization via electrostatic interactions to form flexible helical filaments with a 15 nm diameter.^46–48^ Mixing with ubiquitin chains causes P62 polymers to phase separate into globular structures, called P62 bodies or droplets, which can bind to LC3 and ultimately deliver cargo to the lysosome. Binding of the UBA domain of P62 to the ubiquitin chain is decisive for the formation of the P62 bodies, and the binding is regulated by phosphorylation. The unphosphorylated UBA domain of P62 has a low affinity for ubiquitin, but phosphorylation at S407 or S403 strongly increases the binding affinity.^48,49^ Through ubiquitination at K420 of P62, the recruited kelch-like ECH-associated protein 1 (Keap1)-Cullin-3 complex facilitates the production of P62 bodies.^50^ Ubiquitination of P62 disrupts homodimerization of the P62 UBA domain, leading to the release of the UBA domain to bind ubiquitinated cargos for selective autophagy.^51^

NBR1 is a P62-like receptor with functional domains very similar to P62, but NBR1 has an extra amphipathic α-helical J (JUBA) domain that can bind to lipid membranes.^52,53^ P62 is not required for NBR1 degradation by autophagy. However, under stress conditions, NBR1 and P62 associate with one another through their respective PB1 domains to form hetero-oligomers, thereby promoting the co-localization of NBR1 and P62.^53,54^ In order to activate the KEAP1-nuclear factor erythroid 2-related factor 2 (NRF2) pathway, NBR1 drives the formation of P62 droplets. NRF2 then increases P62 transcription while continually regulating P62 levels via a positive feedback mechanism.^55^

NDP52 is a multifunctional autophagy adapter composed of an N-terminal SKIP carboxyl homology (SKICH) domain, a central coiled-coil domain, and a C-terminal ubiquitin-binding zinc finger (ZF).^56^ NDP52 recognizes ubiquitin chains through its C-terminal binding ZF domain, thereby recruiting ubiquitinated mitochondria. An atypical C-LIR motif, located between the central coiled-coil region and the SKICH domain, interacts with LC3/GABARAP proteins to initiate mitophagy.^38^ Residue 140 of NDP52 is a key regulator of NDP52/LC3C binding, facilitating the production of autophagosomes to drive efficient mitophagy.^57^ Notably, during PINK1-Parkin mitophagy, ATG8 recruitment and selectivity are not dependent on the LIR motif of NDP52.^58^ When NDP52 is recruited to damaged mitochondria, it is oxidized and forms disulfide-linked conjugates on the damaged mitochondria, which promotes the association of its SKICH domain with RB1 inducible coiled-coil 1 (FIP200, one of the important components of unc-51 like autophagy activating kinase 1 (ULK1) complex) and consequently exposes the membrane binding site within the CC domain of FIP200.^59^ This then induces the recruitment of ATG8-positive phagophores by the ULK1 complex.^60^ As a result, additional NDP52 is recruited to the complex through LIR-mediated interactions, forming an ATG8-dependent positive feedback loop to amplify mitophagy signaling.^58^

TAX1BP1 and NDP52 are evolutionarily related and have functional domains very similar to NDP52.^61^ OMM protein ubiquitination causes TAX1BP1 recruitment to depolarized mitochondria, which subsequently recruits LC3 through its LIR region to form autophagosomes to degrade depolarized mitochondrial. Furthermore, TAX1BP1 can recruit autophagosomes through a LC3-independent pathway.^42,62^ When ATG8-lipidation is impaired, TAX1BP1 is recruited to P62-ubiquitin condensates by NBR1. The SKICH domain of TAX1BP1 binds to FIP200 and becomes the main driver for FIP200 recruitment. In P62-NBR1-TAX1BP1 ubiquitin condensates, TAX1BP1 clusters FIP200 to induce local autophagosome formation, thereby replacing the requirement for lipidated LC3. This process provides an alternative mechanism for cells lacking the lipidation machinery.

OPTN that is ubiquitously expressed in cells contains coiled-coil domains, leucine zipper (LZ), a short linear LIR, and Ub-binding domain in ABIN proteins and NEMO (UBAN) and ZF domains.^56^ OPTN is attracted to different mitochondrial regions, in contrast to P62, which is uniformly recruited to damaged mitochondria. OPTN interacts with TANK-binding kinase 1 (TBK1) through its N-terminal coiled-coil domain. TBK1 phosphorylates a number of sites in OPTN when damaged mitochondria are marked with ubiquitin chains. Phosphorylation at Ser 473/513 in the UBAN domain and Ser 177 near the LIR enhances binding of OPTN to ubiquitin and ATG8, respectively.^63,64^ A pathogenic E478G mutation in UBAN domain makes OPTN less able to bind to ubiquitin, thereby inhibiting mitophagy. Furthermore, the LZ motif in OPTN can form a complex with ATG9A vesicles, contributing to the de novo synthesis of autophagosomal membranes independently of the LC3 pathway.^65^ In an artificial liquid-liquid phase separation (LLPS) system, ATG9A-containing vesicles assemble only with the OPTN-Ub LLPS, but not with the NDP52-Ub LLPS or P62-Ub LLPS, suggesting that ATG9A assembly is specific to OPTN.

Studies about these five autophagy receptors were knocked out by CRISPR-Cas9 in cells indicated that only NDP52 and OPTN are primarily essential for mitophagy, which may be due to the function of NDP52 and OPTN in growing the isolation membrane to amplify mitophagy through an NDP52/OPTN-Ulk1-ATG8-NDP52/OPTN-dependent positive feedback loop, as NDP52 and OPTN have lower binding affinity to all ATG8 family proteins compared to P62 and NBR1.^66^ In addition, Parkin can participate in LC3-independent mitophagy. Rab protein, as a small guanosine triphosphatase (GTPase), regulates intracellular membrane trafficking in eukaryotic cells.^67^ Recent research has demonstrated that Rab participates in mitophagy when RABGEF1 is activated. RABGEF1, a guanine nucleotide exchange factor (GEF) of endosomal Rab proteins, can be recruited to damaged mitochondria through ubiquitin binding downstream of Parkin, which then directs the downstream Rab5 and Rab7 to the damaged mitochondria to promote ATG9-mediated vesicle assembly.^68^ Although we have a preliminary understanding of the mechanisms of the action of autophagy adapters in mitophagy, how autophagy adapters avoid redundant roles and achieve unique spatiotemporal expression requires further investigation.

#### TFEB in PINK1-Parkin‐mediated mitophagy

TFEB is an important member of the MITF/TFE family of basic helix-loop-helix leucine zipper (bHLH-Zip) transcription factors.^69^ Nezich et al. found that under the action of ATG5 and ATG9, TFEB underwent Parkin-mediated nuclear translocation to activate mitophagy, indicating that TFEB is a transcriptional regulator of mitophagy.^23^ Subsequently, Ivankovic et al. found that mitochondrial uncoupler carbonyl cyanide 3-chlorophenylhydrazone (CCCP) mediated the PINK1-Parkin pathway in neuroblastoma SH-SY5Y cells to promote the translocation of TFEB to the nucleus, elevating P62 expression and enhancing mitophagy.^70^ Mutation Q311X in Parkin hinders the degradation of PARIS (Parkin interacting substrate), leading to massive aggregation of PARIS protein, which in turn inhibits the nuclear translocation signal of peroxisome proliferator-activated receptor gamma coactivator-1 α (PGC-1α)-TFEB and impairs mitochondrial degradation.^71^ In addition, TFEB regulates Parkin expression. In a carbon monoxide (CO)-induced TFEB nuclear translocation experiment, knockdown of TFEB using siRNA significantly decreased the amount of Parkin recruited to mitochondria, although the expression of PINK was not affected. This finding suggests that TFEB can act as an upstream activation signal of Parkin but not PINK1 to recruit Parkin to mitochondrial fragments, initiating mitophagy.^72^ However, the regulatory mechanism of TFEB’s interaction with PINK1-Parkin signals needs further investigation.

#### DUBs in PINK1-Parkin‐mediated mitophagy

Ubiquitination is a reversible post-translational modification because DUBs can remove ubiquitin from ubiquitinated substrates. At present, the role of E3 ubiquitin ligase Parkin and ubiquitin in mitophagy is well understood, but the regulatory role of DUBs in mitophagy is relatively less studied. USP30, anchored in the OMM, is a mitochondrial DUB. It cleaves Lys 6- and Lys 11-linked multimers assembled at mitochondrial Parkin in response to mitochondrial damage. Overexpression of USP30 promotes deubiquitination on damaged mitochondria, preventing Parkin-mediated mitophagy. USP30 can also directly deubiquitinate MFN2 and TOMM20 to delay the recruitment of Parkin to mitochondria and subsequent mitophagy.^73^ On the other hand, reducing USP30 activity strengthens the degradation of mitochondria in neurons.^74^ USP30 inhibitors reduce the threshold for mitophagy induction and stimulate stress-induced mitophagy.^75,76^ USP8,^77^ USP15,^78^ USP33,^79^ and USP36,^80^ have also been reported to regulate PINK1-Parkin-mediated mitophagy in a positive or negative manner. Nevertheless, it should be noted that most current data are obtained from studies focusing on HeLa cells overexpressing Parkin or Drosophila. Little is known about whether DUB and DUB inhibitors regulate mitophagy in vivo. A recent study reported that ubiquitination of the vast majority of Parkin targets was unaffected in USP30 knockout cells.^81^ Moreover, phosphorylation mediated by PINK1 hinders the enzymatic activity of USP30, which further complicates the study of the mechanism of USP30.^82^ Nevertheless, we need further researches to elucidate the role of DUBs in mitophagy.

### PINK1-Parkin-independent mitophagy

#### E3 ubiquitin ligases in mitophagy

##### ARIH1

**Fig. 3:**
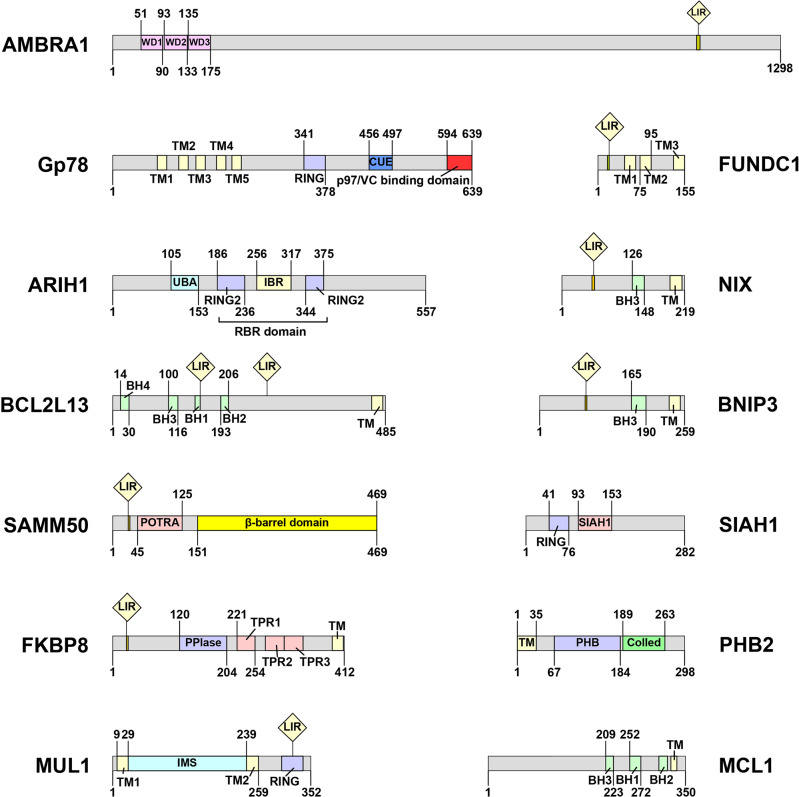
Schematic representation of the domains of proteins involved in the PINK1-Parkin-independent mitophagy

**Fig. 4:**
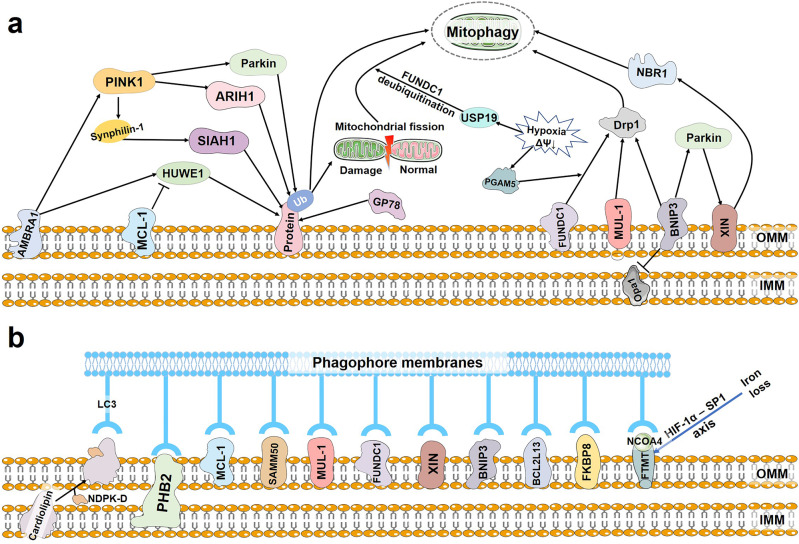
PINK1-Parkin-independent mitophagy. **a** BNIP3 inhibits Opa1 and promotes Drp1, which together induce mitochondrial fragmentation and promote the separation of damaged mitochondria. Meanwhile, BNIP3 recruits Parkin to mitochondria, activating mitophagy. NIX is a substrate of Parkin. After ubiquitination by Parkin, NIX recruits NBR1 to the mitochondria to degrade damaged mitochondria via mitophagy. Upon induction of hypoxia or loss of mitochondrial membrane potential, USP19 promotes deubiquitination of FUNDC1, which promotes mitochondrial fission, leading to mitophagy. In addition, PGAM5 increases FUNDC1-Drp1 complex binding. FUNDC1 dephosphorylation and Drp1-mediated mitochondrial fission together promote mitophagy. Upon mitochondrial depolarization, AMBRA1 is rapidly recruited to OMM to promote PINK1 stability. PINK1 can activate Parkin, ARIH1 and SIAH1 to ubiquitinate OMM proteins, thereby regulating mitophagy. GP78 also ubiquitinates OMM proteins. MUL1 can regulate mitophagy through Drp1 SUMOylation. In addition, AMBRA1 recruits HUWE1 to ubiquitinate OMM proteins. MCL-1 inhibits the recruitment of HUWE1. **b** Cardiolipin, PHB2, MCL-1, SAMM50, MUL-1, FUNDC1, NIX, BNIP3, BCL2L13 and FKBP8 can individually bind to LC3 to mediate mitophagy. Loss of iron leads to impairment of HIF1α degradation, enhancing expression of FTMT via the HIF1α ‐ SP1 axis. Interaction of OMM-localized FTMT with NCOA4 increases the co-localization of FTMT with LC3, thereby promoting mitophagy

Ariadne RBR E3 ubiquitin protein ligase 1 (ARIH1) is an E3 ligase, which belongs to the same RING-in between-RING (RBR) family as Parkin (Fig. 3).^83^ ARIH1 and Parkin are structurally very similar, with the main difference being their expression patterns. Parkin is highly expressed in neuronal cells but is frequently downregulated in cancer cells. On the contrary, ARIH1 is highly expression in cancer cell lines and pluripotent stem cells.^84^ In cancer cells, PINK1 activates ARIH1, and then ARIH1 regulates mitophagy by ubiquitinating OMM proteins in damaged mitochondria (Fig. 4a). ARIH1-dependent mitophagy requires PINK1 and ubiquitination of mitochondrial proteins. However, it does not ubiquitinate any known Parkin substrates (such as MFN2, NPD52, and OPTN), suggesting that ARIH1 has different targets from Parkin.^83^

##### SIAH1

Siah E3 ubiquitin protein ligase 1 (SIAH1) is a RING-type E3-ubiquitin ligase that participates in the mitophagy pathway through the PINK1-synphilin-1 (synuclein alpha interacting protein)-SIAH-1 complex (Fig. 3).^85^ The recruitment of synphilin-1 to mitochondria by PINK1 relies on its direct interaction with synphilin-1 and is independent of PINK1 kinase activity. This is a significant way to distinguish the PINK1-synphilin-1-SIAH-1 pathway from PINK1-Parkin-mediated mitophagy. Synphilin-1 then mobilizes SIAH1 to ubiquitinate mitochondrial proteins, recruiting LC3 as well as the lysosome marker Lamp1 to the mitochondria to initiate mitophagy (Fig. 4a).^86^ Simultaneous treatment with sorafenib (a protein kinase inhibitor) and glucose restriction inhibits hepatocellular carcinoma by impairing SIAH1-mediated mitophagy, suggesting that this pathway may have therapeutic potential in hepatocellular carcinoma.^86^

##### MUL1

As an E3 ubiquitin ligase embedded in the OMM with a RING finger domain facing the cytoplasm, mitochondrial E3 ubiquitin protein ligase 1 (MUL1) has many of the same mitochondrial substrates as Parkin, like dynein-related protein 1 (Drp1) and MFN (Fig. 3).^87,88^ MUL1 can regulate mitochondrial morphology and stimulate mitochondrial fission through Drp1 SUMOylation and MFN2 ubiquitination. Mitochondrial fission is believed to promote mitophagy. Overexpression of MUL1 compensates for PINK1 or Parkin loss in PD, rescuing the PINK1 and parkin mutant-induced phenotypes in dopaminergic neurons and muscles, indicating that MUL1 acts in parallel to the PINK1-Parkin pathway.^89^ Notably, MUL1 acts upstream of PINK1 and promotes PINK1 stability, inducing mitophagy independently of mitochondrial depolarization.^90^ In addition, MUL1 conjugation to UBE2E3 (ubiquitin-conjugating enzyme E2 E3) can bind GABARAP, but not LC3 (Fig. 4b).^91^ These observations indicate that MUL1 functions as both a ubiquitin ligase and a mitophagy receptor.

##### HUWE1

HECT, UBA and WWE domain containing E3 ubiquitin protein ligase 1 (HUWE1), one of the large HECT family members, is involved in the short-life proteins degradation. Similar to Parkin, HUWE1 can ubiquitinate mitophagy-related proteins and participate in mitophagy.^92^ K6-linked Ub chain is an important recognition ubiquitin chain of mitophagy, and HUWE1 can assemble K6-linked Ub chains to initiate mitophagy by ubiquitinating MFN (Fig. 4a). On the other hand, HUWE1 was reported to be a critical E3 ubiquitin ligase for ATG101, promoting ATG101 degradation.^93^ ATG101 is an element of the ULK1 complex. Its degradation suppresses mitophagy and inhibits cancer cell survival. The outcomes of these two studies indicate that the precise role of HUWE1 in mitophagy is determined by the substrates it ubiquitinates.

##### GP78

Glycoprotein 78 (GP78) is an endoplasmic reticulum (ER) membrane–anchored E3 ubiquitin ligase involved in ubiquitination and degradation of various proteins (Fig. 3).^94^ Under normal circumstances, mahogunin RING finger 1 (MGRN1) interacts with and ubiquitinates GP78, thereby targeting GP78 for proteasomal degradation to maintain low levels of GP78.^95^ Mitochondrial depolarization induced by CCCP disrupts the interaction between MGRN1 and GP78, prevents ubiquitination and degradation of GP78 and leads to high GP78 levels, which ubiquitinates MFN1 to promote mitochondrial fission and subsequent mitophagy (Fig. 4a).^94^

#### Autophagy receptors in mitophagy

##### BNIP3 and NIX

BCL2/adenovirus E1B 19 kDa interacting protein 3 (BNIP3) is an OMM protein belonging to the BH3 only Bcl-2 protein family and it is present in various types of cells.^96^ BNIP3 is involved in diverse cellular processes, including, but not limited to, apoptosis, mitochondrial dysfunction, and mitophagy. The structure of BNIP3 includes a characteristic C-terminal transmembrane (TM) domain and a large complex N-terminal region (Fig. 3).^97^ Normally, BNIP3 is expressed as an inactive monomer in the cytosol. In response to stress signals (such as hypoxia), BNIP3 forms a stable homodimer via its C-TM domain and anchors to the OMM.^98^ Deletion of the transmembrane domain disrupts dimer formation and results in defective mitophagy, suggesting that homodimerization of BNIP3 is crucial for mitophagy.^99^ The N-terminal region of BNIP3 contains a LIR motif (‘18-WxxL-21’) enclosed by two phosphorylated serine residues that are Ser17 and Ser24. Phosphorylation of Ser17 and Ser24 is critical for BNIP3 binding to GABARAPL2 and LC3B.^100^ ULK1-mediated phosphorylation of BNIP3 on Ser17 promotes mitophagy. Additionally, ULK1 increases BNIP3 stability through reducing its proteasomal degradation.^101^ In addition, C-Jun N-terminal kinase 1/2 (JNK1/2) and protein phosphatase 1/2a (PP1/2 A) phosphorylate and dephosphorylate BNIP3 at Ser 60/Thr 66, respectively, under hypoxia.^102^ JNK1/2 and PP1/2 A regulate the level of mitophagy by stabilizing and destabilizing BNIP3, respectively, through the ubiquitin-proteasome pathway. Although it is unclear whether the aforementioned enzymes can directly regulate proteasome function, these observations suggest that the level of BNIP3 phosphorylation, rather than the total protein level, is more important for the activation of mitophagy.

NIX is highly homologous to BNIP3 and belongs to the BH3 only Bcl-2 protein family as well. Like BNIP3, NIX contains a C-terminal TM domain and a LIR motif (Fig. 3). Mutation of Ser212 in the C-terminus of NIX impairs its homodimer formation, lowering LC3A-NIX recognition and mitophagy.^103^ On the other hand, phosphorylation at Ser34/35 in the LIR motif of NIX enhances its affinity, increasing recruitment of autophagosomes to mitochondria.^104,105^ The significance of NIX in mitophagy was firstly demonstrated in the generation of mature erythrocytes.^13,106,107^ The expression of NIX is upregulated in terminally differentiated erythrocytes. NIX-mediated mitophagy contributes to the clearance of mitochondria and completes the transition from reticulocytes to mature erythrocytes. Lack of NIX causes defective mitochondrial clearance in reticulocytes, leading to compensatory expansion of erythroid precursors, anemia, and erythroid myeloid hyperplasia. Mitochondrial clearance in NIX^-/-^ reticulocytes can be rescued by highly expression of BNIP3. It is worth noting that there is no amino acid sequence in the minimal essential region of NIX that interacts with LC3.^108^ Mitochondrial clearance in reticulocytes is reduced but not completely blocked in the absence of ATG7, an important protein in the LC3 pathway.^109^ These findings indicate that NIX-mediated mitophagy can occur in a LC3-independent pathway. Furthermore, NIX-mediated mitophagy is required for retinal ganglion cell differentiation and somatic cell reprogramming into induced pluripotent stem cells.^110,111^ Although NIX is recognized to play an irreplaceable role in various cellular processes, how and when NIX is activated in these processes remains to be elucidated.

Unlike Parkin, BNIP3 or NIX do not appear to play an important part in the elimination of depolarized mitochondria in cells, while some data shows that they can improve Parkin-mediated mitophagy and compensate for the absence of functional Parkin. According to the report, BNIP3 inhibits optic atrophy 1 (Opa1)-mediated mitochondrial fusion and promotes the translocation of Drp1 to mitochondria as well.^112,113^ Together, they promote mitochondrial fragmentation and facilitate the separation of damaged mitochondria. Meanwhile, BNIP3 inhibits the proteolytic cleavage of PINK1 kinase by interacting with PINK1, resulting in the accumulation of PINK1 on the OMM, which promotes the recruitment of Parkin to mitochondria and activates mitophagy to clear damaged mitochondria.^114^ NIX is a substrate of Parkin. After being ubiquitinated by Parkin, NIX recruits NBR1 to mitochondria to promote mitophagy (Fig. 4a).^115^ In addition, NIX can compensate for deficiencies in the Parkin pathway. Despite the loss of functional Parkinson’s disease, Koentjoro et al. demonstrated an asymptomatic homozygous carrier of the Parkin mutation who did not develop Parkinson’s disease in her seventh decade.^116^ In a follow-up study, they found that cells from the asymptomatic carrier showed NIX-mediated mitophagy and preserved mitochondrial function compared with cells from Parkin-related PD patients.^117^ Consistently, pharmacological induction of NIX restores mitophagy and mitochondrial function in cell lines derived from Parkin-related PD patients. These findings suggest the crosstalk between different mitophagy mechanisms, but their interplay remains to be further investigated.

##### FUNDC1

FUNDC1 is a OMM protein which is expressed ubiquitously. Human FUNDC1 consists of 155 amino acids containing three highly hydrophilic conserved α-helical segments, including the cytosolic N-terminus, the OMM transmembrane region and the C-terminal region (Fig. 3).^118^ Under hypoxia-induced or carbonyl cyanide p-trifluoromethoxyphenyl-hydrazone (FCCP)-induced stress, FUNDC1 interacts with LC3B via its LIR motif located in the cytoplasmic exposed N-terminal region and acts as a mitophagy receptor to initiate mitophagy.^14,119^ Mutation or deletion of the LIR motif of FUNDC1 impairs FUNDC1-mediated mitophagy. FUNDC1 or hypoxia-induced mitophagy is significantly inhibited in cells deficient in ATG5, but not in ATG6/Beclin-1 deficient cells, suggesting that FUNDC1-mediated mitophagy depends on ATG5.^14,119^

Regulation of FUNDC1 phosphorylation and dephosphorylation is key for its interplay with LC3 and the subsequent regulation of mitophagy. AMP-activated protein kinase (AMPK), a key protein in sensing cellular energy changes, induces the recruitment of autophagy initiation molecule ULK1 to mitochondria under energy depletion.^120–122^ Recruited ULK1 phosphorylates FUNDC1 at Ser17, promoting the interaction between FUNDC1 and Lys49 of LC3B and then mitophagy.^119,123^ LC3B’s side chain undergoes structural rearrangemen to accommodate phosphorylation of FUNDC1, thereby serving as a sensor for the FUNDC1 phosphorylation. In contrast, the side chain of FUNDC1 is extended when it is phosphorylated by Src kinase at Tyr18, which interferes with the hydrophobic pocket of LC3B and disrupts their interaction.^119,123^ In addition, FUNDC1 is phosphorylated by casein kinase 2 (CK2) at Ser13, blocking the LC3B-FUNDC1 interaction, as the backbone carbonyl group and the side chain of Ser13 form hydrogen bonds with the side chain of Arg10 in LC3B. After induction of hypoxia or loss of mitochondrial membrane potential, phosphorylation at Tyr 18 and Ser13 of FUNDC1 undergoes conformational modification through dephosphorylation, facilitating the interaction between FUNDC1 and LC3B and leading to mitophagy. PGAM family member 5 (PGAM5) acts as a positive regulator of mitophagy by dephosphorylating FUNDC1 at Ser13 in a multimeric form.^124,125^ Unlike Tyr18, dephosphorylation of Ser13 does not significantly change the binding affinity of FUNDC1 to LC3B, indicating that the phosphorylation state of Tyr18 in FUNDC1 might act as a critical switch for mitophagy mediated by FUNDC1.

FUNDC1 is closely related to mitochondrial fission, mitophagy and mitochondrial fusion. A dysfunctional mitochondrion must be segregated from the mitochondrial network through mitochondrial fission or other unknown mechanisms before being cleared by mitophagy. The phosphorylation state of FUNDC1 can regulate its interaction with Drp1 or Opa1 to modulate mitochondrial fission or mitochondrial fusion and affect mitophagy. Phosphorylation of FUNDC1 at Ser13 promotes its interaction with Opa1 and reduces its interaction with Drp1, inhibiting mitochondrial fission.^126^ PGAM5 dephosphorylates Ser13 of FUNDC1, disassembles the FUNDC1-Opa1 complex, and increases the formation of the FUNDC1-Drp1 complex. FUNDC1 dephosphorylation and Drp1-mediated mitochondrial fission jointly promote mitophagy. Furthermore, FUNDC1 interacts with the ER membrane protein calnexin at the ER-mitochondrion contact site during early hypoxia. USP19, a mitochondria-associated ER membrane (MAM) protein, accumulates at the ER-mitochondrial contact site, promotes localization of FUNDC1 to MAM, and deubiquitinates FUNDC1 at K119. This induces Drp1 oligomerization and GTPase activity to promote mitochondrial fission, facilitating mitophagy (Fig. 4a).^127^

##### BCL2L13

Bcl2 like 13 (BCL2L13) is an OMM‐anchored transmembrane protein containing four BH domains and two LIR motifs. BCL2L13 is integrated into the OMM through its C-terminal TM domain, with the C-terminus and N-terminus exposed in the IMM space and the cytosol, respectively (Fig. 3).^96^ Early studies found that through its unique C-terminal extension, BCL2L13 activates caspase-3 and releases mitochondrial cytochrome c to induce apoptosis.^128^ Later, Murakawa et al. found that BCL2L13 is a mammalian ATG32 homolog.^129^ Under Parkin-independent conditions, BCL2L13 recruits LC3B to the OMM by interacting with LC3B via a conserved LIR sequence (Fig. 4b). Bcl2-L-13 recruits LC3B followed by or in coincidence with ULK1 complex recruitment, forming the LC3B/ULK1/BCL2L13 complex to induce mitophagy.^130^ It has been reported that the LIR domain in BCL2L13 selectively binds to GABARAP, GABARAP-L, and LC3C to promote mitophagy, while the Q277C/Q278I mutation in the LIR domain enhances its binding to LC3B, indicating that BCL2L13 can bind different LC3/GABARAP family proteins according to varying conditions during the induction of mitophagy.^131^

##### FKBP8

FK506-binding protein 8 (FKBP8, also called FKBP 38) is a newly identified mitophagy receptor in the OMM, containing a TM domain at the C-terminus and a LIR motif at the N-terminus. FKBP8 is anchored in the OMM through its TM domain, with a cytosolic N-terminus (Fig. 3). Under stress conditions (e.g., CCCP treatment, iron depletion or hypoxia), the N-terminal LIR motif of FKBP8 has a strong affinity for LC3A and can recruit lipidated LC3A to damage mitochondria and promote mitophagy in a Parkin-independent manner.^132^ After recruiting LC3A, FKBP8 translocates from mitochondria to the ER, avoiding degradation by autophagosomes. In addition, a LIR motif-like sequence (LIRL) in FKBP8 can bind to Opa1 to mediate mitochondrial fragmentation, which, together with LC3 recruitment mediated by the LIR in FKBP8, is necessary for FKBP8-mediated mitophagy (Fig. 4b).^133^ It is worth mentioning that under hypoxic stress, FKBP8 also binds to FUNDC1. However, the precise function of this interaction in mitophagy is still elusive.

##### AMBRA1

Autophagy/Beclin-1 regulator-1 (AMBRA1) is an active molecule in Beclin-1-regulated autophagy, which is previously identified as a pro-autophagic protein (Fig. 3).^134^ AMBRA1 is rapidly recruited to the OMM during mitochondrial depolarization and then interacts with the ATAD3A complex to promote PINK1 stability.^135,136^ Accumulation of PINK1 facilitates PINK1-Parkin-mediated mitophagy to clear damaged mitochondria efficiently. In addition, AMBRA1 can induce mitophagy independently of the Parkin pathway, in which the E3 ligases HUWE1 and IKKα kinase play important roles.^137^ AMBRA1 recruits HUWE1 to mitochondria, which ubiquitinates OMM proteins (mainly MFN2), targets AMBRA1 to the proteasome and generates signals for phosphorylating Ser1014 in AMBRA1, flanking its LIR motif (Fig. 4b). Then, IKKα kinase phosphorylates Ser1014, which facilitates the LIR motif in AMBRA1 to interact with LC3/GABARAP to activate mitophagy.^137,138^

##### MCL-1

As a member of pro-survival BCL-2 family located in the OMM and matrix, MCL-1 has a unique role in maintaining mitochondrial homeostasis (Fig. 3).^139^ MCL-1 overexpression inhibits HUWE1 recruitment to mitochondria and delays AMBRA1-mediated mitophagy.^140^ During AMBRA1-mediated mitophagy, MCL1 is phosphorylated by GSK-3 at Ser159 and is accompanied by HUWE1-dependent MCL1 degradation. These observations show that MCL-1 is involved in ubiquitin-dependent mitophagy. MCL-1 contains three canonical LIR motifs at its C-terminus and functions as a mitophagy receptor.^16^ After oxygen-glucose deprivation, MCL-1 promotes mitochondrial fragmentation. It also induces mitophagy and facilitates damaged mitochondrial clearance by interacting with LC3A on autophagosomes via its LIR motif (Fig. 4b). UMI-77, known as a crucial anti-apoptotic protein, enhances the interaction between MCL-1 and LC3A, increasing mitophagy. Mitophagy induced by UMI-77 ameliorates cognitive decline and amyloid pathology in the APP/PS1 mouse model of Alzheimer’s disease. However, mutations in the three LIR motifs of MCL-1 do not entirely abrogate mitophagy, suggesting that mechanisms other than the LC3 pathway may also contribute to MCL-1-promoted mitophagy.^141^ In addition, the interaction between MCL-1 and BNIP3 increases during the early stage of hypoxia and under FCCP treatment, suggesting that MCL-1 may promote mitophagy through BNIP3. In conclusion, MCL-1 can regulate mitophagy through various pathways. Understanding how MCL-1 switches among different pathways under physiological and pathophysiological conditions is crucial.

##### SAMM50

Sorting and assembly machinery component 50 (SAMM50), one of the important components of the SAM complex on the OMM, interacts with mitochondrial contact site and cristae organizing system (MICOS) complexes to regulate mitochondrial cristae stability (Fig. 3).^142^ SAMM50-mediated piecemeal mitophagy can continuously replace “worn out” SAM and MICOS complexes to maintain mitochondrial homeostasis. Compared with programmed mitophagy (e.g., mitochondrial removal in erythrocytes or paternal sperm) and stress-induced mitophagy (e.g., PINK1-Parkin pathway activates), piecemeal mitophagy, as a form of basal mitophagy, enables the turnover of specific mitochondrial components by targeting them to lysosomes for degradation.^142,143^ SAMM50 binds to ATG8 through the LIR motif in its N-terminal region and interacts with P62, thereby delivering the SAM and MICOS complex proteins that need to be cleared to lysosomes to maintain mitochondrial integrity (Fig. 4b). Exhaustion of the SAMM50 leads to abnormal mitochondria, reduces ATP production, and increases ROS levels.^144^ In addition, during the transition of cell metabolism from glycolysis to oxidative phosphorylation (OXPHOS), a SAMM50-mediated increase in piecemeal mitophagy elevates the activity and strain on the MICOS proteins and cristae, which help maintain the steady state of mitochondrial networks to provide sufficient ATP.^142^

##### FTMT

As a mitochondrial iron storage protein, mitochondrial ferritin (FTMT) owns ferroxidase activity and is involved in iron loss-mediated mitophagy. Although how iron loss mediates mitophagy remains elusive, it is linked with stabilization of the hypoxia-responsive transcription factor HIF1α and is unrelated to the PINK1-Parkin pathway.^145,146^ Iron loss impairs HIF1α degradation, upregulating FTMT expression through the HIF1α‐specific protein1 (SP1) axis. Interaction of OMM-localized FTMT with nuclear receptor coactivator 4 (NCOA4, a specific cargo receptor for ferritin) increases the co-localization of FTMT with LC3, thereby promoting mitophagy (Fig. 4b).^146,147^

##### PHB2

PHB2 is an IMM mitophagy receptor that binds to PHB/PHB1 to form an alternating tetrameric new gene (RING)-finger domain‐like complex (Fig. 3).^96^ When mitochondria are depolarized, PHB2 binds to PARL to prevent it from directly processing PINK1 in the IMM; meanwhile, PHB2 combines with PGAM5 to maintain the long-chain form of PGAM5, which transiently links to full-length PINK1 to retain PINK1 in the OMM (Fig. 2).^148^ Together, they stabilize PINK1 in the OMM. PHB2 knockdown activates PARL to cleave the long chain of PGAM5 and PINK1, reducing PINK1. Stabilized PINK1 phosphorylates and activates Parkin, leading to proteasome-dependent OMM rupture and subsequent exposure of PHB2. The exposed PHB2 then binds to LC3 via its LIR motif, enhancing PINK1-Parkin-mediated mitophagy (Fig. 4b).^15^ In addition, PHB2 also mediates mitophagy independently of PINK1-Parkin. Mitochondrial aurora kinase A (AURKA) can phosphorylate Ser39 in PHB2, leading to the forming of a tripartite complex of AURKA and MAP1LC3 to induce mitophagy.^149^ PHB2-mediated mitophagy plays an essential role in paternal mitochondrial clearance. Sperm-derived mitochondria accumulate after paternal PHB2 inactivation. However, the exact underlying mechanism remains elusive.

##### Cardiolipin

Cardiolipin (CL) is a unique phospholipid in IMM characterized by a glycerol backbone linked to two phosphatidyl lipids.^150^ CL interacts and stabilizes respiratory chain proteins which include ATP synthase, complexes IV, III, and I. In normal conditions, CL is highly asymmetrically distributed between the OMM (3%) and IMM (97%), forming a gradient in response to changes in mitochondrial homeostasis.^151,152^ When mitochondrial are damaged by rotenone (inhibition of complex I) or CCCP, CL binds to the hexameric membrane spacer protein NDPK-D (also called NM23-H4) and is then externalized from the IMM to the OMM, increasing the amount of CL on the OMM.^152,153^ Elevated OMM CL is then recognized by LC3A/B, leading to CL-mediated mitophagy (Fig. 4b). High interaction with CL is allowed by the unique A14 and K18 residues in the N-terminal region of LC3A.^154^ Knockdown of CRLS1 (responsible for de novo CL synthesis) or scramblase-3 (responsible for CL translocation to the OMM) apparently reduces CL and mitophagy signaling on the OMM.^155^ Interestingly, compared to CCCP-treated HeLa cells, damaged primary neurons had a higher fold in externalized mitochondrial CL.^153^ This observation is consistent with the finding that translocation of Parkin in response to CCCP treatment is diminished or delayed in primary neurons, suggesting a cell type-dependent threshold for externalized mitochondrial CL to participate in specific mitophagy pathways.^156,157^

## Mitochondrial biogenesis and mitophagy jointly maintain mitochondrial homeostasis

The main mitochondrial function is the oxidation of molecules and coupled phosphorylation to generate ATP. Meanwhile, mitochondria produce ROS through chain reactions in energy metabolism. Normally functioning mitochondria clear excess ROS in time to maintain ROS at a low level, which is conducive to cell proliferation. When mitochondria are damaged, clearance of ROS is impaired, resulting in high levels of ROS and cell apoptosis. Hence, the effective removal of damaged mitochondria by mitophagy without harming healthy mitochondria is crucial (Table 1). Mitochondria are always dynamic, changing shapes and sizes through continuous fission and fusion. During fission and fusion, MFN1 and MFN2 distributed in the outer membrane of mitochondria together with Opa1 in the IMM regulate mitochondrial fusion, whereas Drp1 mainly regulates mitochondria division.^158^ Damaged mitochondria can cause asymmetric fission to form two daughter mitochondria with distinct membrane potentials, one depolarized and the other fully polarized. Depolarized mitochondria are then cleared by mitophagy to preserve normally functioning mitochondria.^159^ However, mitophagy decreases the number of mitochondria and thus the energy provided to the body. The increase in AMP/ATP and NAD^+^/NADH ratios activates mitochondrial biogenesis timely.^160,161^ The balance of mitophagy and mitochondrial biogenesis is necessary to maintain mitochondrial homeostasis.

**Table 1:** An overview of PINK1-Parkin-independent mitophagy regulators and their physiological functions

Properties	Protein or Lipid	Mitochondrial localization	Mitophagy inducers	Regulators	Autophagic interactors	Physiological Functions	References
E3 Ubiquitin Ligases	ARIH1	Cytoplasm Nuclear	CCCP	PINK1 ↑	−	Regulate mitophagy by ubiquitinating of OMM proteins in damaged mitochondria	^83,84^
E3 Ubiquitin Ligases	SIAH1	Cytoplasm Nuclear	−	PINK1 ↑ Synphilin-1 ↑ Sorafenib ↓ Glucose restriction ↓	LC3	Regulate mitophagy by ubiquitinating of OMM proteins in damaged mitochondria	^85,86^
E3 Ubiquitin Ligases	MUL1	OMM	Selenite	ULK1 ↑	GABARAP	Rescue the PINK1 and Parkin mutant phenotypes in dopaminergic neurons and muscle Participate in the elimination of paternal mitochondria in mouse embryos	^368,369^
E3 Ubiquitin Ligases	HUWE1	Cytoplasm	−	−	−	Regulate mitophagy by ubiquitinating proteins	^92,93^
E3 Ubiquitin Ligases	GP78	Endoplasmic reticulum membrane	CCCP	MGRN1 ↓	LC3	Ubiquitinate Mfn1, thereby promoting mitochondrial fission to induce mitophagy	^94,95^
Autophagy Receptors	BNIP3	OMM	Hypoxia	FOXO3 ↑ HIF1A ↑ MA-5 ↑ ULK1 ↑ JNK1/2 ↑ PP1/2 A ↓	LC3B GABARAPL2	Activate mitophagy during hypoxia or increased oxidative stress	^96,98,100–102^
Autophagy Receptors	NIX	OMM	Hypoxia High OXPHOS activity	HIF1A ↑	GABARAPL1 LC3A LC3B LC3-independent	Participate in retinal ganglion cell differentiation and somatic cell reprogramming to induced pluripotent stem cells Participate in generation of mature erythrocytes	^13,106,110,111^
Autophagy Receptors	FUNDC1	OMM	Hypoxia FCCP	ULK1 ↑ SRC ↓ CK2 ↓ PGAM5 ↑ USP19 ↑ MIR137 ↓	LC3B	Involved in hypoxia-mediated mitophagy	^14,119,120,123,125,127^
Autophagy Receptors	BCL2L13	OMM	CCCP	ULK1 ↑	LC3B LC3C GABARAP GABARAP-L	Promote mitophagy of damaged mitochondria in a Parkin-independent manner	^130,131^
Autophagy Receptors	FKBP8	OMM	Starvation Iron depletion Hypoxia	RHEB↓	LC3A	Escape from the mitochondria to the endoplasmic reticulum after recruitment of LC3A to avoid degradation	^96,132,133^
Autophagy Receptors	AMBRA1	OMM	FCCP Mitochondrial depolarization	IKKα kinase ↑ HUWE1 ↑ MCL1 ↓ GSK3B ↑	LC3 GABARAP	Promote Parkin-mediated mitophagy and Parkin independent mitophagy	^137,140^
Autophagy Receptors	MCL-1	OMM	Oxygen-glucose deprivation FCCP Early stage of hypoxia	GSK3B ↓	LC3A	Participate in the regulation of mitophagy through various pathways Ameliorate cognitive decline and amyloid pathology in the APP/PS1 mouse model of Alzheimer’s disease	^16,139–141^
Autophagy Receptors	SAMM50	OMM	Under normal circumstances	−	ATG8 protein	SAMM50-mediated piecemeal mitophagy maintain mitochondrial homeostasis Promote cell metabolism from glycolysis to OXPHOS	^142–144^
Autophagy Receptors	FTMT	OMM	Iron loss	HIF1α ↑	LC3	Participate in iron loss-mediated mitophagy	^145,146^
Autophagy Receptors	PHB2	IMM	CCCP Oligomycin Antimycin	AURKA ↑	LC3B	Involved in removal of paternal mitochondria	^15,148,149^
Autophagy Receptors	Cardiolipin	OMM IMM	Rotenone CCCP	CRLS1 ↑ PLSCR3 ↑	LC3A LC3B	Redistribute to the outer membrane of damaged mitochondria where it promotes mitophagy	^151,153,155^

The PGC-1α-NRF-1/2-transcription factor A, mitochondrial (TFAM) pathway regulates mitochondrial biogenesis. The PGC-1α mRNA levels are increased dramatically in thermogenic tissues in response to cold, leading to increased content of mitochondrial DNA (mtDNA).^162^ PGC-1α overexpression significantly promotes content of mtDNA and mitochondrial in myocytes of transgenic mice.^163,164^ PGC-1α is now recognized as a master regulator of mitochondrial biogenesis, coordinating essential proteins expression for mitochondrial biogenesis via transcription factors NRF1/2. It is of note that mitochondrial proteins are increased before changes in PGC-1α expression.^165^ This rapid response may be due to activation of rather than increased expression of PGC-1α. NRF1/2 binds to the promoter regions of various mitochondrial genes, including TFAM. Deletion of the N-terminal fragment of NRF1 or NRF2 deficiency blocks the effects of PGC-1α on mitochondrial biogenesis, suggesting that NRF1/2 act downstream of PGC-1α.^164,166^ TFAM belongs to the high mobility group box domain family and is crucial for mtDNA replication. TFAM binds upstream of the transcription start site, ensuring the unwinding and flexing of mtRNA required for binding of mitochondrial RNA polymerase to the mtDNA promoters.^167,168^ In addition, TFAM contains two DNA-binding sites that compact mtDNA through loop formation and cross-strand binding for packaging in nucleoids.^169^ The compact form of TFAM can significantly increase the number of fully compacted mtDNA molecules, participating in mtDNA storage. The loose form of TFAM is involved in active replication and transcription. Thus, TFAM function is crucial for mitochondrial biogenesis. Furthermore, since TFAM expression parallels the parameters of mitochondrial biogenesis, TFAM is widely accepted as a marker of mitochondrial biogenesis.^170^ However, the uncertainty of TFAM levels as a biogenesis marker has recently been reported because it fails to accommodate the expression of mtDNA-encoded polypeptides and mtDNA quantities.

**Fig. 5:**
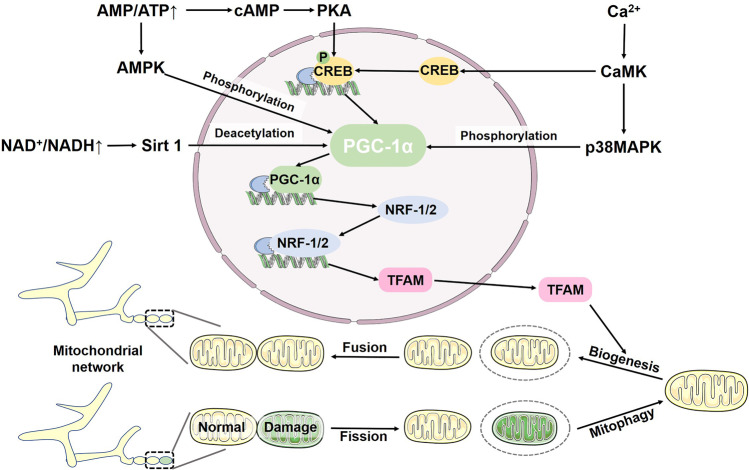
Mitochondrial biogenesis and mitophagy jointly maintain mitochondrial homeostasis. Mitochondrial biogenesis is regulated by the PGC-1α-NRF-1/2-TFAM pathway. Elevated AMP activates AMPK, which directly phosphorylates PGC-1α, increasing expression of PGC-1α and TFAM. In addition, AMP can be converted to cAMP, which regulates PGC-1α through the cAMP-PKA-CREB pathway. Ca^2+^ stimulates CaMK to phosphorylate p38 MAPK. Additionally, CaMK can stimulate PGC-1α via CREB. In response to NAD^+^, Sirt1 deacetylates PGC-1α to activate PGC-1α

Several signaling cascades can regulate mitochondrial biogenesis by affecting the PGC-1α-NRF-1/2-TFAM pathway. Of these, the AMP/ATP ratio, Ca^2+^ levels and NAD^+^/NADH ratio are the most relevant. Elevated AMP activates AMPK that directly phosphorylates PGC-1α and increases the expression of PGC-1α and TFAM.^160^ In addition, AMP could be converted to cyclic AMP (cAMP) by adenylate cyclase (AC), which regulates PGC-1α through the cAMP-PKA-CREB pathway to promote mitochondrial biogenesis.^171^ Ca^2+^ stimulates calcium/calmodulin-dependent protein kinase (CaMK), which in turn phosphorylates p38 mitogen-activated protein kinase (p38 MAPK), which increases the activity and expression of PGC-1α and then mitochondrial biogenesis.^172–174^ Additionally, CaMK can stimulate PGC-1α through CREB, suggesting that CREB may be involved in the Ca^2+^-dependent mitochondrial biogenesis. Sirtuin 1 (Sirt1) deacetylates PGC-1α to activate PGC-1α and consequently promote mitochondrial biogenesis in response to NAD^+^ (Fig. 5).^161^ Notably, deacetylation of PGC-1α involves Ca^2+^, AMPK, and Sirt1, implying that the major mitochondrial biogenetic stimuli may be interconnected.^175^

## Mitophagy and disease

### Mitophagy and neurodegenerative diseases

Neurodegenerative disease is a general term for a set of diseases caused by chronic progressive degeneration of nerve tissue, which is the most common nervous system disorder in the aged and is featured by selective degeneration and loss of central neurons. Most patients often have symptoms, including cognitive decline, memory loss, and speech and daily activity impairments. Common neurodegenerative diseases include Parkinson’s disease (PD), Alzheimer’s disease (AD), Huntington disease (HD) and Amyotrophic lateral sclerosis (ALS). Neurons demand a lot of energy, and abnormalities in structure and function of mitochondria can cause neuronal degeneration. Emerging research shows that mitophagy is closely associated with neurodegenerative diseases.^176–178^

#### Parkinson’s Disease (PD)

PD is caused by the degeneration of dopaminergic neurons in the substantia nigra. The main manifestations are increased muscle tension throughout the body, slow movements, and dull facial expressions, often accompanied by static tremors.^179^ The occurrence of PD is correlated with mutations in some key proteins, like α-Synuclein (α-syn),^180^ leucine-rich repeat kinase 2 (LRRK2),^181^ protein DJ-1 (DJ-1),^182^ F-Box protein 7 (Fbxo7)^183^ and vacuolar protein sorting 35 (VPS35).^184^ Deng et al. found that in the PD animal model, mitochondria were enlarged and edematous, suggesting the existence of mitophagy disorder.^185^ α-syn has then been reported to induce neuronal death by increasing mitophagy in mutant A53T mice. In yeast cells, the toxicity of α-syn can be transmitted through mitophagy mediated by Sir2, the yeast homolog of mammalian SIRT1.^186,187^ Later studies reported that α-syn,^188^ LRRK2,^189^ DJ-1,^190^ Fbxo7^191^ and VPS35^192^ all affect the occurrence of PD through pathways related to Parkin-mediated mitophagy. For example, overexpression of mutant A53Tα-syn leads to p38 MAPK activated, which phosphorylates Parkin at serine 131 and subsequently causes mitochondrial dysfunction and neuronal death.^188^ Additionally, these proteins mediate mitophagy through other mechanisms. G2019S mutation of LRRK2 can slow the removal of Miro from OMM and then delay mitochondrial arrest and mitophagy.^193^ The mutation also increases phosphorylation of RAB10 at threonine 73 in PD patients, thereby decreasing accumulation of OPTN on depolarized mitochondria and impairing depolarization-induced mitophagy.^194^ LRRK2 kinase inhibitors correct G2019S-induced mitophagy deficiency independently of the PINK1-Parkin pathway.^195^ These observations suggest that the PD-related proteins can affect PD progression by altering mitophagy, which can be corrected by pharmacological modulators, opening a new avenue for future PD therapy.

#### Alzheimer’s Disease (AD)

AD is brought on by neurons loss and synapses and is featured by abnormal deposition of β-amyloid (Aβ) and accumulation of hyperphosphorylated Tau protein (pTau).^196^ Its main clinical feature is degeneration of the cerebral cortex, resulting in memory loss and cognitive dysfunction. Mitochondrial dysfunction induced by mitophagy is one of the cores of AD pathogenesis. Mitochondrial dysfunction, as reported, can precede the accumulation of Aβ deposits.^197,198^ Normally functioning mitochondria can reduce aberrant amyloid precursor protein (APP) processing and the buildup of Aβ plaques.^199^ Ye et al. first revealed Parkin-mediated enhancement of mitophagy in mutant hAPP neurons and AD patient’s brains.^200^ Additionally, cytosolic Parkin in the AD patient’s brains is depleted as the disease progresses, leading to abnormal accumulation of PINK1. Parkin overexpression effectively restores mitophagy. Besides, it reduces the accumulation of defective mitochondria.^201^ Restoration of mitophagy favors inhibition and reduction of Aβ plaques, elimination of tau hyperphosphorylation, and prevention of cognitive dysfunction.^177,202^

Abnormal accumulation of tau protein also affects the function of mitophagy. The N-terminal tau fragment can stably bind Parkin and cytosolic ubiquitin-c-terminal hydrolase L1 (UCHL-1), which causes abnormal recruitment of Parkin and UCHL-1 to mitochondria and subsequent undue mitochondrial removal and synapse loss, leading to the occurrence of AD.^203^ Accumulation of tau can also induce mitophagy impairment by directly inserting into mitochondrial membrane, resulting in increased mitochondrial membrane potential, reduced Parkin recruitment, and then increased neuronal toxicities.^204^ Interestingly, according to reports, tau does not suppress Parkin translocation to mitochondria by regulating mitochondrial membrane potential.^205^ Instead, Parkin translocation is prevented by tau’s abnormal interaction with Parkin in the cytoplasm, which occurs significantly at 48 h but not 24 h after transfection under CCCP treatment.^205^ These reports suggest that during its early accumulation phase, Tau can bind to specific proteins, like UCHL-1, to recruit Parkin to mitochondria, removing damaged mitochondria. Once excessive mitophagy occurs, Tau can prevent Parkin translocation and inhibit mitophagy via attaching to Parkin in the cytosol or increasing the membrane potential. However, further research is required to delineate the molecular mechanism through which Tau regulates mitophagy.

#### Huntington Disease (HD)

HD is a neurological disorder linked to loss of motor coordination. The etiology of HD is related to mutations in the gene encoding Huntington (Htt), mainly characterized by an extra stretch of polyglutamine (polyQ) repeats at the N-terminus of the htt protein.^206^ Expanded polyQ repeats interact abnormally with glyceraldehyde-3-phosphate dehydrogenase (GAPDH, a dehydrogenase implicated in glycolysis), reducing the activation of GAPDH-induced mitophagy.^176^ Furthermore, expanded polyQ repeats interfere with the formation of ULK1/PtdIns3K and interaction with OPTN/NDP52, thereby affecting the trigger of mitophagy and the recruitment of LC3 to mitochondria.^207^ Defects in mitophagy result in the accretion of damaged mitochondria and increased cell demise, promoting the progression of HD. Thus, prompt and effective elimination of damaged mitochondria is one of the keys. Enhancing the PINK1-Parkin-mediated mitophagy pathway can improve the integrity of mitochondria and neuroprotection in HD.^208^ Notably, hyperactivation of mitophagy also promotes the development of HD. VCP, a class II member of ATPase, can bind to mutant Htt and accumulate in mitochondria, causing overactivation of PINK1-Parkin-dependent mitophagy and ultimately neuron death.^209^

#### Amyotrophic Lateral Sclerosis (ALS)

ALS is caused by motor neuron degeneration and is mainly manifested as progressive muscle weakness and atrophy, of which about 10% are familial.^210^ Mutations in multiple genes contribute to the progression of ALS. SOD1 (a gene encoding a Cu/Zn superoxide dismutase) was the first gene to be discovered.^211^ Mutant SOD1 blocks the reverse transport of mitochondria in neurons. It also aggregates and sequesters OPTN, inhibiting the formation of mitophagosomes and then mitophagy flux.^212,213^ These combined effects bring about the buildup of damaged mitochondria at axon terminals, reducing neuronal survival. According to reports, the Parkin-mediated mitophagy pathway is activated in the ALS mutant SOD1 mouse model. PINK1-Parkin double knockout results in the accretion of damaged mitochondria, which lowers mitochondria function and causes degeneration of ALS neuromuscular junctions.^214^ Conversely, Palomo et al. reported that in the SOD1-G93A mouse model, ablation of the Parkin gene reduces mitochondria depletion and delays ALS progression.^178^ However, to date, there has been no plausible explanation for these discrepancies.

### Mitophagy and cardiovascular diseases

#### Cardiac differentiation

Due to its particularity, the heart needs to constantly contract and relax, making it an organ with a high energy demand. Therefore, healthy mitochondria are essential for the heart, and normal mitophagy is conducive to the normal differentiation of the heart. Yang et al. reported that tamm41 regulates the differentiation of heart valves through PINK1-Parkin-mediated mitophagy, and lack of tamm41 leads to abnormal heart valves.^215^ Gong et al. demonstrated that mitochondrial normal maturation is directed by Parkin-mediated mitophagy in perinatal mice cardiomyocytes, which is beneficial for the maturation of cardiac metabolism.^216^ Interestingly, during differentiation of cardiac progenitor cells, the mitochondrial network remodeling does not require the PINK1-Parkin-mediated mitophagy pathway; instead, it is mediated by BNIP3L/NIX and FUNDC1.^217^ These results indicate that the normal differentiation of the heart requires the participation of multiple mitophagy pathways.

#### Cardiomyopathies

Abnormal mitophagy impairs mitochondria function, leading to insufficient energy in myocardial cells and resulting in a series of myocardial diseases. Billia et al. observed mitochondrial swelling and abnormal mitochondrial function in PINK1-deficient mice, which eventually brings about left ventricular dysfunction as well as pathological cardiac hypertrophy.^218^ Song et al. found that in adult mice, ablating Drp1 interrupts mitochondrial fission and significantly elevates Parkin levels, enhancing Parkin-mediated mitophagy.^219^ Excessive activation of mitophagy causes mitochondrial depletion and fatal cardiomyopathy. Ablation of Parkin reduces Drp1 deficiency-induced mitophagy and delays cardiomyopathy. In addition, insulin-like growth factor receptor II (IGF-IIR) promotes phosphorylation of Drp1 by activating extracellular signal-regulated kinase (ERK), resulting in excessive mitochondrial division. IGF-IIR signaling also triggers the formation of Rab9-dependent autophagosomes. Excessive mitochondrial division enhances phagocytosis of damaged mitochondria by the Rab9 pathway, ultimately reducing cardiomyocyte survival.^220^ Although the expression levels of Drp1 in the above two experiments are different, the outcome is essentially due to the hyperactivation of mitophagy, which phagocytoses too many mitochondria and affects the energy supply in cardiomyocytes, leading to their damage. How to ensure that the expression level of Drp1 is beneficial to the protection of cardiomyocytes needs further study. In addition, Bhandari et al. found that lack of Parkin in Drosophila causes mitochondria dysmorphology and depolarization in cardiomyocytes, resulting in dilated cardiomyopathy.^221^ Unlike mice, Parkin redundant genes are deficient in Drosophila, which more underscores the importance of PINK1-Parkin-mediated mitophagy for removing damaged mitochondria in cardiomyocytes.

#### Heart failure

When the heart is chronically stressed, it can cause mitochondrial damage in myocardial cells. If mitophagy fails to clear damaged mitochondria in time, it can lead to cardiomyocyte apoptosis and even heart failure. Under stress conditions, activation of mitochondrial protease OMA1 induces the cleavage of Opa1 from the long chain (L-Opa1) to the short chain (S-Opa1), which suppresses mitochondrial fusion and fragments mitochondria, resulting in the death of necrotic cells, fibrosis as well as ventricles remodeling.^222^ Wang et al. found that AMPKα2 promotes phosphorylation of PINK1 at Ser495 and activates the PINK1-Parkin pathway to increase myocardial mitophagy, thereby removing damaged mitochondria in myocardial cells under stress and preventing the progression of heart failure.^223^ In addition, heart failure may occur when the heart is under abnormal hemodynamic pressure overload for a long time. In a mice model of increased pressure load caused by transthoracic aortic constriction, Shirakabe et al. found that mice develop heart failure after 30 days of long-term observation.^224^ During this period, mitophagy is activated transiently ~3–7 days after transthoracic aortic constriction because of the translocation of Drp1 to mitochondria. Drp1 knockout abolishes mitophagy, which aggravates the dysfunction of mitochondria and the development of heart failure after transthoracic aortic constriction. Further studies found that the mitophagy pathway activated after transthoracic aortic constriction is primarily ULK-, Rab9-, Drp1-, and receptor interacting protein 1 (Rip1)-dependent alternative mitophagy, instead of Parkin-, LC3-, ATG7-dependent mitophagy.^225^ TAT-Beclin1 treatment reactivates traditional or alternative mitophagy to defer the development of heart failure.^225^

#### Myocardium aging

The time of aging determines the time for cardiomyocytes to function, and mitophagy performs a significant part in removing abnormal mitochondria and inhibiting aging-related diseases occurrence. Ma et al. found that in the rat cardiomyocyte model, the accumulation of PINK1 and Parkin in the mitochondria and the decrease of mitochondrial ubiquitination occur in the process of cell aging, which leads to decreased mitophagy and increased cell damage and aging.^226^ Antioxidant treatment to cardiomyocytes significantly improves mitophagy response, alleviating cell injury. Furthermore, Gao et al. found that Parkin activates the Parkin-TBK1-P62 signaling pathway to promote mitophagy and reduce cardiac senescence by inducing polyubiquitination of the K63 link of TBK1 in mice.^227^

### Mitophagy and lung diseases

#### Acute lung injury

Acute lung injury (ALI) is the damage of capillary endothelial cells and alveolar epithelial cells induced by different injury factors, leading to alveolar edema and diffuse pulmonary interstitial. As research progressed, mitophagy has been found to perform a significant part in ALI.^228–230^ In ALI caused by lipopolysaccharides (LPS)-induced sepsis in mice, Zhang et al. found that LPS exposure decreases mitochondrial membrane potential in lung tissue.^230^ Parkin is recruited from the cytoplasm to the mitochondria, which reduces MFN2 protein expression and increases Drp1 protein expression. The PINK1-Parkin-mediated mitophagy pathway is activated and enhanced to remove excess mitochondria and then increase apoptosis, which can be effectively suppressed by mitochondrial division inhibitor 1 (Mdivi-1). Mdivi-1 inhibits mitophagy activation by reducing the expression of Drp1, which decreases lung cell apoptosis and protects the lung tissue from oxidative stress in LPS-treated rats.^229^ These findings indicate the participation of mitophagy during the process of lung tissue damage in ALI. Interestingly, Letsiou et al. analyzed the levels of PINK1 and Parkin after 18 h of LPS induction. Then they found that PINK1 expression is increased while Parkin expression is decreased.^228^ The reduction in Parkin expression alleviates mitophagy, promotes mitochondrial fusion repair, and protects mitochondria.^231^ This suggests that the downregulated expression of Parkin may be a compensatory mechanism to prevent excessive mitophagy from clearing too many mitochondria.

Under normal circumstances, mitochondrial biogenesis and mitophagy are in a dynamic balance to ensure appropriate control of mitochondrial quality and quantity. In ALI, mitophagy is increased to remove damaged mitochondria, which disrupts proper mitochondria homeostasis and excessively eliminates mitochondria. In this situation, increased mitochondrial biogenesis can maintain mitochondria homeostasis and protect lung tissue in ALI. In an LPS exposure model, MAP kinase 3 (MKK3) knockout upregulates regulatory factors expression involved in mitochondrial biogenesis and enhances mitochondrial biogenesis to ensure normal quality and quantity of mitochondria, resulting in a reduction in lung injury and an improvement of survival in mice.^232^ In another animal model of ALI induced by Staphylococcus aureus, mitophagy and mitochondrial biogenesis occur simultaneously, which increases the turnover of mitochondria and reduces the death of type II alveolar cells.^233^ These findings indicate that lung tissue can be protected against increased mitophagy by enhancing mitochondrial biogenesis, opening up a new treatment option for ALI.

#### Chronic obstructive pulmonary disease

Chronic obstructive pulmonary disease (COPD), which is characterized by persistent respiratory symptoms and airflow limitation, is a chronic inflammatory disease. One of the causative factors of COPD is exposure to cigarette smoke. Exposed to cigarette smoke for a long time causes abnormal structure and function of mitochondria and accumulation of mitochondrial fragments, suggesting impairment of mitophagy.^234^ Nevertheless, the function of mitophagy still remains controversial when it comes to the development of COPD. Parkin level is decreased in lung homogenate of COPD patients and is positively associated with FEV1/FVC ratio in lung function tests.^235^ Decreased Parkin expression can inhibit mitophagy induced by cigarette smoke extracts (CSE), increasing ROS production and HBEC cell aging. This suggests that cell senescence caused by insufficient Parkin-mediated mitophagy may be one of the momentous factors in COPD progression. In contrast, Mizumura et al. found that enhanced mitophagy can lead to programmed cell death, which is involved in the development of COPD.^236^ This process is dominated by PINK1 but does not require Parkin, indicating that it is independent of the PINK1-Parkin pathway. These two seemingly contradictory reports may indicate two different pathways involved in the development of COPD. The concentration of CSE used in the two studies is 1% and 20%, respectively, suggesting the main pathway engaged in the development of COPD might be associated with the degree of mitochondrial damage. When exposed to a high concentration of CSE (20%), mitochondrial damage exceeds the compensation threshold. Massive PINK1 accumulation on OMM leads to hyperactivation of mitophagy, resulting in programmed cell death. In the face of low concentrations (1%) of CSE, while transient mitochondrial damage is within the range of compensatory regulation, long-term damage reduces cytosolic Parkin pool, leading to insufficient mitophagy and the development of COPD. Although the mechanism of Parkin reduction is unclear, it may involve PINK1-mediated proteasomal degradation.^237^

#### Idiopathic pulmonary fibrosis

Idiopathic pulmonary fibrosis (IPF) is a progressive chronic interstitial lung disease.^238^ It is featured by increased extracellular matrix deposition, repeated damage to the alveolar epithelium, and ultimate pulmonary fibrosis or even organ death. Changes in mitophagy are related to the progression of IPF pulmonary fibrosis, but its effect depends on the type of lung cells. For alveolar macrophages, mitophagy is increased during fibrosis to counteract apoptosis. Meanwhile, enhanced mitophagy favors the expression of macrophage-derived transforming growth factor-β1 (TGF-β1).^239^ TGF-β1 is one of transforming growth factors for fibroblast differentiation and promotes pulmonary fibrosis. For fibroblasts, mitophagy is reduced during pulmonary fibrosis, leading to insufficient ROS clearance, the differentiation of myofibroblasts and the formation of fibroblast foci.^240,241^ For type II alveolar epithelial cells, as IPF lungs age, the expression of PINK1 decreases, which causes mitochondrial swelling and defects in mitophagy, promoting changes in lung fibers.^242,243^ It is worth mentioning that these three types of lung cells influence each other in the progression of fibrosis. For example, macrophage-derived TGF-β1 can restrain PINK1 expression in fibroblasts to inhibit mitophagy and promote lung fibrosis; whereas it induces epithelial cells to produce ROS, causing cell death and pulmonary fibrosis.^244,245^

### Mitophagy in acute kidney injury

Acute kidney injury (AKI) is a clinical syndrome that results from a sharp reduction of renal function in a short period of time due to assorted causes, some of which may be related to mitophagy. Sepsis, as we all know, is one of the most common etiologies of AKI. Takasu et al. analyzed kidney tissue from patients with sepsis and found that renal tubular cells have coarse or finely dispersed vacuoles, indicating swollen mitochondria and lysosomes and abnormal mitophagy.^246^ Subsequently, in the septic AKI mouse model induced by cecal ligation and puncture (CLP), the PINK1-Parkin-mediated mitophagy pathway is activated in the early stage of CLP.^247^ Further studies indicate that PINK1-Parkin deficiency reduces mitophagy and worsens kidney injury and renal tubular cell apoptosis.^248^ It has been reported that both sepsis-induced and contrast media (CI) -induced (another common cause) AKI activate mitophagy.^249^ In a mice model of CI-induced AKI, mitophagy is triggered in renal tubular epithelial cells (RTECs) exposed to CI, which protects RTEC from apoptosis and tissue damage by lowering mitochondrial ROS and inflammatory NLRP3 activation.^250^ In addition, Lei et al. found that mTOR inhibitor rapamycin enhances mitophagy by inhibiting mTOR and then reduces renal tubular cell damage induced by CI.^251^ However, this approach to upregulate mitophagy remains controversial in the field, as inhibition of mTOR promotes cell apoptosis and inhibits proliferation of renal tubular cells, which is detrimental to recovery from AKI. However, it is valuable noting that mitophagy plays a crucial protective role in AKI regardless of the cause.

Cisplatin is a chemotherapeutic drug that is effective in treating many types of cancers. However, its use remains limited due to its pronounced nephrotoxicity.^252^ The underlying mechanism has been extensively studied for many years but remains unclear. Recently, mitophagy was found to be activated in human renal proximal tubule cells treated with cisplatin. Mitophagy inhibited by inhibition of PINK1-Parkin expression leads to mitochondrial dysfunction and increased cell damage, while PINK1-Parkin overexpression protects mitochondrial function and cell damage.^253^ This indicates that mitophagy is involved in the procedure of cisplatin-mediated nephrotoxicity. Consistently, Wang et al. discovered that knockout of PINK1 impairs renal mitophagy, resulting in more severe apoptosis, tissue damage and loss of renal function.^252^ However, it is reported that PINK1 cannot improve cisplatin-induced kidney injury in rats by Zhou et al.^254^ These conflicting results may be due to the varying severity of mitochondrial damage induced by different doses of cisplatin used in animals. Knockout of PINK1 partially reduces mitophagy and rescues cells from apoptosis. On the other hand, in Zhou’s experiments, many autophagosomes and mitophagosomes were found in cisplatin-treated WT rats, indicating excessive mitophagy.^254^ Therefore, when the mitochondrial damage is within the compensation range, activation of mitophagy may effectively eliminate damaged mitochondrial caused by cisplatin. Once mitochondria are excessively damaged, mitophagy is overactivated, leading to cell apoptosis.

To repair damaged renal tubules following kidney injury, surviving renal tubular cells undergo dedifferentiation, proliferation, migration, and redifferentiation to become mature intrinsic cells.^255^ Blockage of renal repair results in renal fibrosis, an eventual common route causes end-stage renal failure. To our knowledge, mitochondrial damage is closely related to abnormal kidney repair.^256^ In unilateral ureteral obstruction (UUO)-renal fibrosis, PINK1 or Parkin deficiency not only leads to insufficient mitophagy but also increases the production and damage of mitochondrial ROS in renal tubular cells after UUO, consequently aggravating renal fibrosis.^257^ The mitophagy activator UMI-77 reduces profibrotic responses in renal tubular epithelial cells by activating mitophagy.^258^ In addition, in the UUO-induced experiments, PINK1 or Parkin deletion led to excessive accumulation of abnormal mitochondria in macrophages, which accelerated the transformation of macrophages into profibrotic /M2 macrophages and the progression of renal fibrosis. These findings imply that mitophagy has an anti-fibrosis effect on renal tubular cells and macrophages, which is beneficial to the repair of kidney injury.^259^ In summary, mitophagy performs a key role in the emergence and AKI repair, and may become a turning point in the prevention and treatment of AKI.

### Mitophagy and liver disease

#### Alcoholic liver disease

Alcoholic liver disease (ALD) is liver damage induced by long-term excessive alcohol intake. It initially manifests as significant hepatic steatosis and eventually develops into serious liver diseases, such as cirrhosis. In humans, alcohol metabolism occurs primarily in the liver.^260^ Heavy drinking or chronic exposure to alcohol stimulation leads to impaired hepatic mitochondrial function, causing liver damage. Mitophagy can prevent alcohol-induced liver damage by clearing abnormal mitochondria.^261,262^ In acute alcohol binge, hepatic mitochondria undergo depolarization due to the metabolic effects of alcohol in a dose-dependent manner.^263^ If mitochondrial depolarization fails to repolarize normally, mitophagy is activated to remove uncoupled and abnormal mitochondria to preserve normal mitochondrial function and avoid cell apoptosis and liver damage. Mitophagy also reduces alcohol-induced hepatic lipid accumulation and steatosis by maintaining normal fatty acid β-oxidation.^264^ Long-term alcohol stimulation triggers continuous and excessive mitochondrial clearance load signals and subsequent mitophagy disorder. As a result, mitochondrial damage-associated molecular patterns are released into the cytosol and extracellular milieu, thereby stimulating inflammation and fibrosis in the liver and promoting ALD development.^260^ This process may be related to the DNA-dependent protein kinase catalytic subunit (DNA-PKcs), a brand-new steward of hepatic mitochondrial homeostasis.^265^ In chronic alcohol induction, DNA-PKcs promotes P53 activation, increases Drp1-related mitochondrial division and simultaneously inhibits FUNDC1-mediated mitophagy. Mitochondrial DNA damage and apoptosis are caused by excessive fission and defective mitophagy, implying that the impact of mitophagy is linked to the amount and time of alcohol consumption and suggesting that manipulation of mitophagy may become a prospective treatment for ALD.^265^

#### Non-alcoholic fatty liver disease

Nonalcoholic fatty liver disease (NAFLD) is a metabolic syndrome with unknown etiology, and its main cause is aberrant lipid buildup in the liver.^266^ Mitophagy favors normal clearance of lipids, and lipid accumulation indicates aberrant mitophagy. Consistently, swollen mitochondria and decreased cristae activity are observed in NAFLD patients.^267^ In a long-term high fat/calorie diet-induced mouse NAFLD model, PINK1-Parkin-mediated mitophagy is increased in the early stage to counteract hepatic lipid accumulation.^268^ Prolonged feeding reduces both mRNA and protein levels of PINK1-Parkin, leading to lipid accumulation and nonalcoholic steatohepatitis.^268^ Activation of mitophagy attenuates hepatic steatosis and NAFLD progression.^267,269^ Therefore, how to restore mitophagy activity during decompensation is the key to the treatment of NAFLD. The expression of Mst1 (a cell survival regulator related to liver regeneration) is upregulated in an animal model of NAFLD. Mst1 knockdown restores Parkin-mediated mitophagy, thereby attenuating liver injury and improving hepatocyte viability.^270^ NAFLD can also be prevented by restoring the Parkin-independent mitophagy pathway. For example, Sirtuin 3 overexpression can protect hepatocytes from apoptosis by promoting BNIP3-mediated mitophagy through the ERK-CREB signaling pathway.^271^ On the other hand, KEAP1 and RING-box protein 1 (Rbx1), two subunits of a cullin-RING ubiquitin E3 ligase complex, are recruited to mitochondria by P62, and then the P62-KEAP1-Rbx1 complex promotes mitochondrial ubiquitination to restore mitophagy.^272^

### Mitophagy in skeletal muscle aging

Skeletal muscle is the driving force of the human motor system, and the key process in skeletal muscle development regulation is myogenesis. During early myogenic differentiation, Drp1-mediated mitochondrial fragmentation and SQSTM1-mediated mitophagy coordinate to clear mitochondria, and then the activation of PGC-1α mediates the biogenesis of mitochondria to produce new mitochondria. Finally, the rapid upregulation of Opa1 completes the reorganization of the mitochondrial network to meet the energy demands after differentiation of primitive myoblasts into mature myotubes.^273^ Inhibition of mitophagy impairs mitochondrial remodeling and myogenic differentiation. It has been reported that MFN2 expression is decreased with age in the skeletal muscle of mice. MFN2 deficiency results in reduced mitophagy and buildup of damaged mitochondria, causing sarcopenia as well as atrophy.^274^ Consistently, Parkin-mediated mitophagy deficiency impairs mitochondrial function and causes muscle atrophy. On the other hand, overexpression of Parkin increases mitochondrial content and prevents the loss of muscle mass and strength that comes with age.^275,276^ Drp1-mediated mitochondrial fission promotes mitophagy to clear dysfunctional mitochondria in aged flight muscles and prolong lifespan in midlife Drosophila.^277^ It is worth mentioning that, unlike the heart and brain, skeletal muscle has regenerative repair capacity due to the presence of satellite cells, but satellite cells’ ability to restore injured muscle descends significantly with age.^278^ Genetic inhibition of mitophagy in satellite cells of young mice leads to premature senescence.^279^ This finding is somewhat surprising because mitophagy was formerly thought to be an effector pathway instead of senescence cause. Meanwhile, it indicates the possibility of slowing satellite cell senescence through modulating mitophagy.

Long-term proper exercise can slow down muscle aging, while mitophagy contributes to muscle aging. However, little is known about the effect of exercise on mitophagy. Studies have shown that AMPK, ULK1 and Drp1 are transiently phosphorylated during or early after (0–1 h) acute exercise and activate mitophagy to clear damaged or dysfunctional mitochondria during the post-exercise recovery period (3–6 h).^280^ Lack of Drp1 reduces muscle endurance and running performance.^281^ On the other hand, Lo Verso et al. found that mitophagy is not involved in sustaining muscle contraction during physical activity, but instead plays a role in damaging contraction.^282^ In a 6-week-long mice voluntary wheel running experiment, Parkin expression in wild type mice is upregulated to promote the degradation of PARIS (Parkin interacting substrate) after 6 weeks of training, thereby promoting PGC-1α expression. The increase in mitochondrial biogenesis and mitophagy promotes mitochondria renewal. The same study also reported that in the face of the same exercise training, exercise-induced mitophagy decreases with the increase in the number of exercises.^283^ Therefore, it is possible that mitophagy activation rely upon the kind and length of exercise or, more precisely, on the energy required for sudden exercise. AMPK judges whether energy is sufficient by sensing changes in cellular energy. If cells can supply sufficient energy to maintain muscle contraction, mitophagy is not activated during post-exercise recovery. If energy is insufficient during recovery, mitophagy is activated and coordinates with mitochondrial biogenesis to renew and produce better-quality mitochondria. This can reduce the activation of mitophagy in the next recovery period after similar exercise intensity. Long-term exercise training renews mitochondria to meet more energy supply and delays the problem of insufficient energy in skeletal muscle caused by aging.

### Mitophagy in cancer

#### Cancer cells

Given that cancer cells are constantly dividing rapidly, they rely on the Warburg effect to obtain sufficient energy through glycolysis rather than mitochondrial OXPHOS production, even under aerobic conditions.^284^ Diminished mitochondrial ROS, decreased total mitochondrial membrane potential per cell and mitochondrial mass are observed in KRAS-mutant pancreatic cancer cells, indicating a reduced mitochondrial network. However, mitochondrial biogenesis-related markers are not significantly changed during the process. On the other hand, mRNA levels of proteins linked to mitophagy (e.g., NIX, P62) are increased, which displays an inverse relationship to mitochondrial content.^285^ These observations indicate that NIX-mediated mitophagy is related to the progression of pancreatic cancer cells. NIX-mediated mitophagy expression reduces the mitochondrial network and increases the conversion of glucose to lactate through glycolysis, providing sufficient energy for cancer cells. In mice, NIX deficiency greatly inhibits tumor growth and prolongs survival. Enhanced mitophagy is also observed in esophageal squamous cell carcinoma and lung cancer.^286,287^ Conversely, deletion or loss-of-function mutations in Parkin are detected in lots of tumors, including glioblastoma, ovarian cancer and breast cancer cells included, which leads to deficient mitophagy and elevated mitochondrial ROS levels.^288–290^ ROS can stabilize HIF-1α and activate glycolysis, promoting the Warburg effect and tumor progression.^291–294^ Therefore, how cancer cells adapt to hypoxic conditions may depend on the cell type.

#### Cancer stem cells

Traditional cancer treatment is to destroy large numbers of proliferating cancer cells. However, this approach often fails to eradicate cancer because cancer stem cells are not removed. Cancer stem cells refer to cancer cells with stem cell properties, which have the ability of “stemness” (including self-renewal, differentiation, proliferation and metastasis potential). Numerous studies reported perinuclear distribution of mitochondria, higher membrane potential, lower quantity of mtDNA, lower intracellular concentrations of ROS and lower oxygen/glucose consumption in cancer stem cells, which allows for better retention of stemness.^295,296^ For example, the perinuclear distribution of mitochondria favors the transport efficiency of ATP; lower glucose consumption indicates that cancer stem cells are relatively quiescent and enter a dormant state to enhance their adaptability and avoid destruction. Since mitophagy is the key to controlling mitochondrial and energy metabolism, it is likely that mitophagy performs an important role in preserving “stemness” characteristic of cancer stem cells. Phosphorylated P53 is degraded with PINK1-dependent mitochondrial clearance, and suppression of mitophagy results in accumulation of phosphorylated P53 in mitochondria.^297^ Excess P53 then translocates to the nucleus. Also, it binds to NANOG promoter to prevent NANOG activation. NANOG is a fundamental transcription factor to maintain the stemness of hepatic cancer stem cells. NANOG inhibition suppresses hepatic cancer stem cells propagation. In addition, mitochondrial dynamics assist mitophagy in completing the “stemness” expression of cancer stem cells. To ensure that the new stem cells remain their properties, mitochondria divide asymmetrically, and healthy mitochondria enter new stem cells.^298^ Overexpression of mitochondrial fission factor (MFF) boosts mitochondrial fission to enhance the stemness and tumor-initiating capacity of liver cancer-initiating cells.^299^ Similarly, in primitive acute myeloid leukemia cells, activating mitophagy needs the expression of mitochondrial fission 1 (FIS1). Depletion of FIS1 attenuates mitophagy, resulting in a severe loss of their ability to self-renew and promotion of myeloid differentiation in Leukemia stem cells.^300^ In addition, the expression of FIS1 also promotes stemness in the lung cancer stem cells of human via mitophagy.^301^ It may offer hope for a cure for cancer if the role of mitophagy in cancer stem cells can be applied therapeutically.

### Mitophagy and immunity

#### Innate immunity

Innate immunity refers to the nonspecific recognition of foreign pathogens or damage-associated molecular patterns (DAMPs), and is the first line of body to protect against pathogen invasion.^302,303^ Upon recognition of a pathogen or DAMPs, the upregulation and release of interferons, cytokines, and chemokines recruit immune cells to contain the infection or injury, triggering an inflammatory response. In PINK1 and parkin KO mice, acute (exhaustive exercise-induced) and chronic (mtDNA mutation-induced) mitochondrial stress induces stimulator of interferon genes (STING, a central regulator of the type I Interferon response to cytosolic DNA)-mediated type I interferon response.^304^ After binding to mtDNA accumulated in the cytoplasm, cyclic GMP-AMP synthase (cGAS) is activated and produces 2'3'-cGAMP, a second messenger, which in turn leads to the dimerization of STING and the activation of downstream TBK1.^305–308^ Activated TBK1 phosphorylates transcription factors in the cytoplasm to promote their nuclear translocate, upregulating the generation of cytokines and interferons. These findings imply that PINK1-Parkin-mediated mitophagy could regulate innate immune responses through the STING pathway.

Inflammasomes are a multiprotein complex assembled by intracytoplasmic pattern recognition receptors, among which the NLRP3 inflammasome, as a vital part of innate immunity, performs a significant impact on the process of body immune response and disease occurrence. Increased ROS and mtDNA induced by mitochondrial damage initiate the inflammatory response by activating NLRP3 inflammasome and caspase-1, promoting the maturation and secretion of the pro-inflammatory cytokines interleukin-1β (IL-1β) and IL-18.^309^ Therefore, mitophagy can negatively regulate the activation of NLRP3 inflammasomes by clearing damaged mitochondria in a timely manner. For example, in LPS-treated macrophages, pro-IL-1α translocates to mitochondria and directly interacts with cardiolipin (CL), interrupting the CL-LC3B-dependent mitophagy in activated macrophages and enhancing activation of NLRP3 inflammasomes.^310^ NLRP3 inflammasome activation causes nuclear factor kappa B subunit (NFKB/NF-κB) to selectively induce SQSTM1 accumulation on mitochondria, thereby initiating mitophagy to exert its anti-inflammatory effect.^311^ Studies have also found that levels of the stress inducible protein senstrin 2 (Sesn2) are upregulated by carbon monoxide. Sesn2 marks damaged mitochondria to mediate SQSTM1 aggregation; on the other hand, it also activates ULK1-specific mitophagy machinery to clear damaged mitochondria, which inhibits the activation of NLRP3 inflammasome and prevents septic shock induced by LPS.^312,313^ The discovery of this intracellular anti-inflammatory mechanism may provide new ideas for anti-infection.

The function of the NLRP3 inflammasome in the clearance of viruses has been widely recognized. Several new studies have reported that viruses can specifically regulate the balance between mitophagy and the NLRP3 inflammasome to promote their replication. Parkin knockout mice display enhanced innate antiviral inflammation and increased viral clearance through increasing mtROS-mediated NLRP3 inflammasome activation. And loss of NLRP3 reverses the boosted antiviral response in Parkin knockout mice.^314^ Enhancing mitophagy appears to be a good option for viruses to avoid early clearance by NLRP3 inflammasomes. Influenza A virus protein PB1-F2 is translocated to mitochondria through TOM40 channels. It reduces mitochondrial membrane potential to induce mitochondrial fragmentation, thereby attenuating the retinoic acid-inducible gene I (RIG-I) signal and disrupting the NLRP3 inflammasome pathway.^315^ Given that RIG-I-like receptors are sensors that initiate and regulate antiviral immunity, reducing their signal impairs clearance of influenza a virus (IAV). However, according to some reports, Ripk2 (receptor interacting protein kinase 2) activates mitophagy by phosphorylation of ULK1. Knockout of Ripk2 in mice impairs mitophagy, enhances NLRP3 activation, and increases mortality upon influenza a virus infection.^316^ It is of note that this is only one manifestation of pathogenicity to host cells after massive propagation of influenza A viruses. Additionally, HIV long terminal repeat region (ssRNA40) can inhibit mitophagy from activating the NLRP3 inflammasome in human primary microglia, promoting neurotoxicity and neurodegeneration by enhancing ROS production.^317^ Injection of berberine into IAV-infected mice alleviates inflammatory lesions in the lungs by activating mitophagy and inhibiting NLRP3 inflammasome activation.^318^

Macrophages are essential immune cells in innate immunity and have plasticity and pluripotency. They can be classified as M1 type (classically activated macrophage) and M2 type (alternatively activated macrophage) based on different activation states and functions. M1 type macrophages are induced by LPS and interferon gamma (IFN γ) signals and produce and release pro-inflammatory cytokines (like TNF α and IL-1β) and cellular byproducts (like ROS, lysosomal enzymes and nitric oxide) to participate in inflammatory reactions and remove pathogens. M2 type macrophages are induced by Th2 type cytokine stimulation in the local microenvironment, mediate the production of anti-inflammatory effects as well as take part in the repair and fibrosis of injured tissues through secreting cytokines, like IL-10.^319^ In addition, due to the different energy requirements of M1/M2 macrophages, mitochondrial metabolism also affects M1/M2 conversion. The highly pro-inflammatory effects of M1 macrophages rely on glycolysis and pentose phosphate pathway and are accompanied by increased glucose uptake. The anti-inflammatory function of M2 macrophages is greatly reliant upon mitochondrial OXPHOS, tricarboxylic acid cycle flux, and fatty acid oxidation.^320^

Recently, macrophages were found to exhibit the M1 type in diabetic nephropathy mice. Similarly, when treated with high glucose, macrophages switch to the M1 type with decreased mitophagy.^321^ Rapamycin-induced enhancement of mitophagy prevents M1 polarization, suggesting that activation of mitophagy can promote the transformation of macrophages to the M2 type. In mice fed a high-fat diet, knockout of ATG5 increases macrophage M1 polarization and inflammation in the liver.^322^ Therefore, it is possible that mitophagy affects mitochondrial metabolism and regulates the phenotype of macrophages. Activation of M1 macrophages inhibits mitochondrial function and OXPHOS, resulting in the inability of M1 macrophages to repolarize to M2.^323^ IL-10 activates mitophagy by inhibiting mTOR signaling, prevents the accumulation of damaged mitochondria induced by LPS stimulation, and simultaneously inhibits glycolytic flux, thereby stimulating macrophage to polarize toward the M2 type.^324^ Exogenous IL-10-stimulated generation of M2 macrophages can promote wound healing by reducing inflammatory response after myocardial infarction (MI).^325^ On the other hand, lack of NIX reduces M1-related pro-inflammatory cytokines expression and glycolysis in M1 macrophages.^110^ Therefore, if and how mitophagy regulates macrophage phenotype polarization is complex and remains unfathomable.

#### Adaptive immunity

Adaptive immunity usually refers to the entire process in which T and B lymphocytes in body are stimulated by “non-self” substances, activate, proliferate, differentiate into effector cells, and generate a sequence of biological effects. Comparing the mitochondrial function of T, B lymphocytes and their subpopulations at various phases of differentiation and activation revealed significant differences in their mitochondrial ROS, mitochondrial membrane potential, mitochondrial mass as well as mitophagy extents.^326^ This finding suggests that mitophagy participates in the development and differentiation of T and B lymphocytes. During primary response to virus infection, CD8^+^ T cells downregulate NIX expression as a reaction to the T cell antigen receptor (TCR) signal, leading to insufficient mitophagy and buildup of depolarized mitochondria, which generates superoxide to activate CD8^+^ T cells.^327^ However, antigen-specific CD8^+^ T cells boost the expression of NIX to remove depolarized mitochondria undergoing contraction state, thereby promoting CD8^+^ T effector memory cells to form. Loss of NIX results in insufficient clearance of depolarized mitochondria, accumulation of HIF1α, altered effector memory cell metabolism (from long-chain to short-chain/branched-chain fatty acid oxidation), and reduced ATP synthesis. Energy deficit inhibits CD8^+^ T cell effector memory to form and decreases recall reaction to cytopathic viral infection. Likewise, knockdown of NIX and BNIP3 causes mitochondrial buildup, decreases mitophagy and enhances fatty acid synthesis, resulting in raised lipid droplets in the cytoplasm and loss of IgG^+^ memory B cells. Inhibition of fatty acid production or silencing necroptosis gene Ripk3 rescues IgG memory in NIX^-/-^BNIP3^-/-^ B cells.^328^ Furthermore, natural killer (NK) cells are innate lymphocytes with adaptive immunity characteristics. Similar to CD8^+^ T cells, the level of mitophagy in NK cells is temporarily downregulated during viral infection.^329^ During the contraction phase, enhancement of mitophagy removes ROS and depolarizes mitochondria, thereby inducing NK cell memory to form following viral infection. Overall, mitophagy is crucial for the differentiation of immune cells into corresponding memory cells. Up to date, we are still facing a stage where the development of various vaccines is stagnant due to the inability to induce powerful memory cells. Many immune diseases cannot be effectively treated because the problem of memory cells cannot be resolved. Strengthening the study of mitophagy on cellular memory can promote progress in the fields of vaccines and immune diseases.

### Mitophagy and metabolic syndrome

Metabolic syndrome refers to the pathological state of metabolic disorder in human body. Obesity and insulin resistance affect metabolic pathways, such as glucose and lipids, and can cause metabolic syndrome. In obese humans or rats fed high fat, the levels of mitophagy-related signals LC3BII, Parkin, FUNDC1, and BNIP3 are decreased, suggesting that mitophagy may be causally related to obesity and insulin resistance.^330,331^ Ablation of FUNDC1 brings about defective mitophagy in anabolic white adipose tissue (WAT), leading to massive ROS generation, which resulting in oxidative stress-driven MAPK activation.^332^ Furthermore, MAPK activation blocks insulin signaling, resulting in insulin resistance in other insulin-responsive organs. Inhibition or ablation of MAPK attenuates obesity and insulin resistance phenotypes.^332,333^ In addition, mitophagy affects the function of catabolic brown adipose tissue (BAT). PINK1 knockout mice exhibit catabolic BAT dysfunction and predispose to obesity.^334^ NLRP3 expression is observed in brown adipocyte precursors (BAPs) of PINK1 knockout (KO) mice. Unexpectedly, typical inflammasome activity in BAPs was not induced by NLRP3 expression. Conversely, it suppresses brown adipocyte-specific genes expression and promotes white adipocyte-specific genes expression, resulting in the differentiation of BAPs into white-like adipocytes. Loss of NLRP3 reverses BAT dysfunction and obesity in PINK1 KO mice. Furthermore, global but not brown adipocyte-specific PINK1 KO mice exhibit insulin resistance, suggesting that mitophagy functions differently in maintaining insulin sensitivity in WAT and BAT.^334^

### Mitophagy in deafness and eye diseases

#### Deafness

Deafness refers to the general term for different degrees of hearing damage caused by organic or functional lesions of auditory organs and auditory conduction pathways, and is commonly seen in hair cell (HC) damage. Aminoglycosides are commonly used antibiotics, but it is toxic to sensory HC. According to some recent studies, defects in the function of mitochondria and excessive buildup of ROS in HC are key mechanisms of aminoglycoside-induced hearing loss.^335,336^ Neomycin represses PINK1 mRNA transcription by activating ATF3 in cochlear HCs and decreases PINK1-Parkin-mediated mitophagy.^336^ Deficiency in mitophagy causes massive dysfunctional mitochondria and ROS accumulation, inducing the death of hair cells and following hearing loss. Restoration of mitophagy using a mitophagy inducer deferiprone and a PINK1 activator kinetin protects HCs from neomycin-induced apoptosis, suggesting that mitophagy activators may serve as a potential drug for protecting HCs from aminoglycoside-induced damage.^336^ In addition, activation of mitophagy can protect against age-related or noise-induced hearing loss.^337,338^ NIX-mediated mitophagy expression maintains cochlear cell homeostasis during aging.^337^ SESN2 deficiency impedes mitophagy and exacerbates noise-induced auditory impairment.^338^ In conclusion, mitophagy may be a new target for the treatment of deafness.

#### Eye diseases

In eye diseases, people becoming aware of the significance of oxidative damage, mitochondrial dysfunction and impaired mitochondrial clearance. A variety of eye diseases are caused by mitophagy. The core mechanism of dry eye is tearing hypertonicity. Oxidative damage to mitochondria and disturbances in energy metabolism are observed in corneal epithelial cells (HCECs) cultured in high osmotic pressure.^339^ Energy disturbance leads to activation of AMPK, which phosphorylates MFF to recruit DRP1 to OMM to initiate mitochondrial fission and mitophagy. Increased mitochondrial fission elevates ROS levels and exacerbates mitochondrial dysfunction, thus forming a vicious circle. In seniors, age-related macular degeneration (AMD) is a primary cause of blindness. Reduced mitophagy and impaired NFE2L2 antioxidant signaling that are observed in the early retinal pigment epithelium (RPE) of AMD can trigger death-resistant epithelial-mesenchymal transition.^340^ Two pathways, mitophagy and NFE2L2 antioxidant signaling, work together to inhibit RPE conversion. Loss of NFE2L2 or PGAM5 leads to decreased mitophagy and mitochondrial turnover in RPE, thereby accelerating RPE senescence.^341,342^ In addition, mitophagy also affects the development of glaucoma, which is featured by progressive damage to retinal ganglion cells (RGCs) and can lead to irreversible blindness. Its major risk factor is elevated intraocular pressure (IOP). In a chronic hypertensive glaucoma model, a compensatory parkin-mediated mitophagy is increased within 3 days to clear damaged mitochondria.^343^ As the cumulative damage of moderate IOP elevation, the compensatory mechanism of mitophagy begins to fail, and mitophagy is impaired with the progressive injury of RGCs. Overexpression of Parkin or deletion of uncoupling protein 2 partially restores mitophagy function under cumulative stress of elevated IOP, thereby protecting RGCs in glaucoma.^343,344^

### Mitophagy in aging

Aging, a biological process, refers to the cell proliferation and physiological functions gradual reduction over time. During aging, the structure and function of mitochondria undergo extensive changes, and generated ROS cannot be effectively cleared, resulting in mitochondrial dysfunction.^345^ Morphologically and functionally, aging in Drosophila and mammals is characterized by increased mitochondrial volume, irregular cristae size and shape, raised ROS production, declined ATP synthesis, as well as reduced mitochondrial numbers. According to an increasing number of studies, mitophagy is essential for the aging process of cells and organisms. Mitophagy has been reported to increase from adolescence to adult before a sharp decrease in elderly animals.^346^ Basal mitophagy levels in flight muscles of 4-week-old Drosophila are approximately tenfold higher than in 1-week-old flies.^347^ In mouse dentate gyrus (a brain region important for memory), mitophagy is greatly reduced between 3 and 21 months of age.^348^ Increased mitophagy is beneficial to slow the progression of cellular senescence and age-related diseases.^237,241,341,349,350^ Overexpression of mutant DRP1-K38A or S637A in PGAM5-deficient cells promotes mitophagy and inhibits retinal pigment epithelial senescence.^341^ Bone morphogenetic protein 4 (BMP4) activates mitophagy and prevents fibroblasts from aging, thereby suppressing the differentiation of fibroblasts to myofibroblasts and consequently alleviating the progression of pulmonary fibrosis.^241^ Furthermore, an enhanced understanding of mitophagy during aging has particular implications for the amelioration of genetic diseases featured by premature aging. In Cockayne syndrome, Hutchinson-Gilford Progeria Syndrome, and Werner syndrome, defects in mitophagy and sustained ROS production have been observed.^351–355^ Activation of mitophagy can resist cellular senescence and apoptosis. As reported, the induction of mitophagy is sufficient to extend lifespan and delay accelerated aging in several model organisms.^346,349,356^ NAD^+^ activates mitophagy to remove damaged mitochondria and restore the mitochondrial network, inhibiting premature aging and prolonging the lifespan. Aging is an inevitable path for every cell and is closely related to diseases in all systems. A proper knowledge of the function of mitophagy in aging is very important for alleviating and preventing the aging process.

## Limitations and development of mitophagy in targeted therapy

**Fig. 6:**
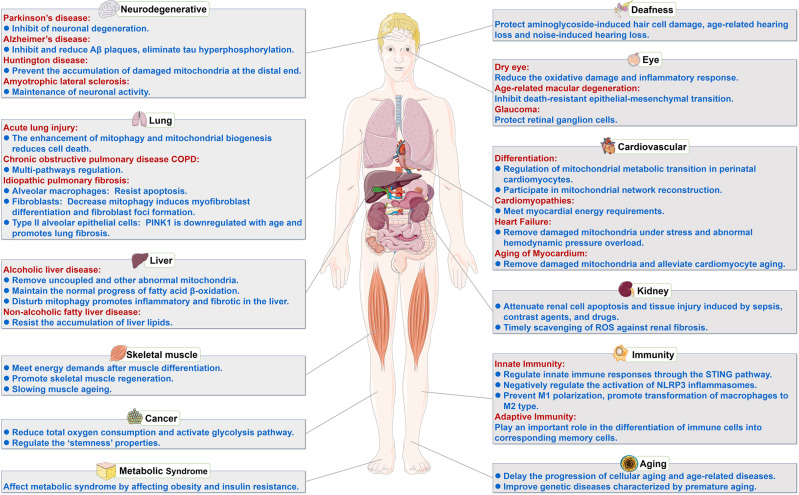
Mechanisms of mitophagy in various diseases

Mitophagy that is too weak or too strong is not conducive to cell survival. Excessive mitophagy leads to over-clearance of mitochondrial and insufficient energy supply, resulting in cell death. On the other hand, weak mitophagy leads to inadequate clearance, causing mitochondrial dysfunction and reduced cell survival. Given the important role of mitophagy in various human diseases (Fig. 6), pharmacological modulation of mitophagy is potentially an effective way to treat mitochondrial-related diseases. With continued investment, more and more drugs are being discovered (Table 2). However, research on mitophagy modulators is still preliminary due to the short overall research time, and most data come from preclinical studies. Before these modulators enter clinical trials or potent new effective modulators are investigated, the following two conditions should be considered.

**Table 2:** Drugs associated with mitophagy modulators

Pharmaceutical	Targeted pathway	Diseases	Physiological functions and disease links	References
Urolithin A	Promote mitophagy	Parkinson’s disease	Inhibit NLRP3 inflammasome activation via promoting mitophagy, protecting against dopaminergic neurodegeneration and neuroinflammation	^365^
6′′′-Feruloylspinosin	Promote the expression of PINK1/Parkin	Alzheimer’s disease	Alleviate beta-amyloid induced toxicity by promoting mitophagy	^370^
UMI-77	Activate PINK1 signaling	Alzheimer’s disease	Ameliorate cognitive decline and amyloid pathologies	^16^
Kaempferol, Rhapontigenin	Activate mitophagy	Alzheimer’s disease	Increased the survival and functionality of glutamatergic and cholinergic neurons, abrogated amyloid-β and tau pathologies, and improved the animals’ memory	^371^
Spermidine	Promote the expression of PINK1-PDR1	Neurodegenerative diseases	Ameliorate the symptoms of neurodegenerative and premature aging diseases by promoting PINK1-PDR1-dependent mitophagy pathway	^372^
Baicalein	Activate FUNDC1 signaling	Cardiac hypertrophy	Attenuate cardiac hypertrophy via suppressing oxidative stress and activating mitophagy	^373^
α-lipoic acid	Activate FUNDC1 signaling	Heart failure	Protect against pressure overload-induced heart failure via FUNDC1 dependent mitophagy signaling	^374^
Ellagic acid	Suppress BNIP3 overexpression	Heart failure	Suppress BNIP3 induced mitochondrial injury and cell death in ventricular myocytes	^375^
Resveratrol	Upregulate the mitochondrial translocation of Parkin	Myocardium aging	Alleviate Senescent-Like Cell Phenotypes by promoting Parkin-mediated mitophagy	^367^
Tetrahydroberberrubine	Promote PHB2 overexpression	Myocardium aging	Promote mitophagy and retard cardiomyocyte senescence	^376^
Oxyberberine, MCTR3	Downregulate PINK1/Parkin signaling	Acute lung injury	Alleviate LPS-induced inflammation in ALI via inhibition of mitophagy	^377,378^
Bone morphogenetic protein 4	Activate PINK1 signaling	Idiopathic pulmonary fibrosis	BMP4 attenuates fibroblast-to-myofibroblast differentiation by reducing impaired mitophagy and cellular senescence in lung fibroblasts	^241^
Quercetin	Promote the expression of SIRT1/PINK1	Kidney fibrosis	Alleviate kidney fibrosis through the SIRT1/PINK1/mitophagy axis	^379^
Tetramethylpyrazine	Promote the expression of PINK1/Parkin	Alcoholic liver disease	Contribute to necroptosis inhibition by facilitating PINK1/parkin-mediated mitophagy	^380^
Cyanidin-3-O-glucoside, Quercetin	Promote the expression of PINK1/Parkin	Non-alcoholic fatty liver disease	Alleviate hepatic steatosis by enhancing PINK1/Parkin-dependent mitophagy	^267,381^
Akebia Saponin D	Promote the expression of BNIP3	Non-alcoholic fatty liver disease	Alleviate hepatic steatosis by promoting BNIP3 induced mitophagy	^382^
Zinc oxide nanoparticles	Promote the expression of PINK1/Parkin	Tongue squamous cell carcinoma	Induce toxicity in CAL 27 oral cancer cell lines by activating PINK1/Parkin-mediated mitophagy	^383^
Aloe gel glucomannan	Promote the expression of PINK1/Parkin	Colon cancer cells	Induce cytotoxic mitophagy through ROS-related PINK1/Parkin pathway	^384^
Ketoconazole	Downregulate cyclooxygenase-2, accumulation PINK1/Parkin	Hepatocellular carcinoma (HCC)	Exacerbate mitophagy to induce apoptosis, thereby inhibiting the growth of HCC	^385^
Flavaglines compound 3	Inhibit PHB2-PARL-PGAM5-PINK1 axis	Cancer	Inhibit PHB2-mediated mitophagy and effectively block cancer cell growth	^148^
Qidan Tiaozhi capsule	Activate AMPK/PINK1-Parkin-mediated mitophagy	Metabolic syndrome	Suppress oxidative stress to treat metabolic syndrome	^386^

First, to be therapeutically targeted, these modulators must specifically target mitochondria to regulate mitophagy. However, the modulators discovered so far have limited specificity for mitophagy because of their low pharmacological specificity for mitophagy targets. It is a logical approach to finding modulators that indirectly regulate mitophagy. However, it may not be suitable for therapeutic use due to the highly potent off-target effects. A recent study reported the first mitophagy-specific degradation product, AUTAC. AUTAC is an autophagy-targeting chimera with a degradation tag (guanine derivatives) and a warhead to offer target specificity.^357^ When AUTAC targets mitochondria, it improves mitochondrial quality by removing damaged mitochondria through a non-PINK1-Parkin pathway. In addition, gene editing is another promising treatment for specifically targeting mitophagy. It is feasible to achieve “gain-of-function” specific transgenes and “loss-of-function” gene ablation to regulate mitophagy temporally and spatially in animal models.^333,358–360^ However, this is only the beginning, many problems remain to be solved.

Second, to ensure that mitophagy is strictly and precisely regulated and to enable a simple and practical assessment of treatment effects, it is critical to find biomarkers that can reliably and specifically detect mitophagy flux. Measurements of LC3-II/I and SQSTM1 are now commonly used to reflect mitophagy flux. Human mitophagy assessment is confined to genetic examination (such as detecting mRNA transcripts of PINK1, Parkin, etc.) or histological analysis (providing tissue photos of small samples of lesions). Although these approaches can reflect changes in mitophagy flux, the outcomes are challenging to interpret. For example, whether the changes are caused by excessive mitochondrial degradation or blockage of mitophagy, and sometimes these changes do not necessarily reflect mitophagy activity or do not specifically represent mitophagy situation. It has been reported that some mitochondria-targeted fluorescent markers, such as mt-Keima and Mito‐QC, can dynamically reflect mitophagy flux.^348,361–363^ MT Keima is a pH-sensitive fluorescent protein targeting the matrix of mitochondria, allowing analysis of mitochondrial degradation in vivo and in vitro by fluorescence microscopy, with pH-dependent excitation spectrum peaks.^348,361,362^ The excitation peak of mt-Keima changes from 438 nm at neutral pH to 550 nm at acidic pH during transport from mitochondria to lysosomes, resulting in dual excitation ratiometric imaging. Mt-Keima has been used to detect mitophagy in mammalian cells and tissues because it can maintain fluorescence in an acidic environment and has resistance to lysosomal degradation. However, due to the characteristics of mt-Keima dual excitation ratiometric imaging, its excitation peaks gradually change as the emission spectra overlap.^361,363^ Interpretation of mitophagy is complicated by spectral overlap. In addition, analysis with the mt-Keima mouse model requires fresh sectioning and immediate visualization of tissues, so it is impossible to detect mitophagy in interest cells that have been immunolabeled in tissues simultaneously.

Mito-QC, a mCherry-GFP-tagged fluorescent marker, is similar to mt-Keima and sensitive to pH. It is fused to the TM domain of the OMM-anchored protein FIS1.^363^ After mito-QC-containing mitochondria (mCherry and GFP positive) are transported to lysosomes, mCherry is robust to acidic pH, whereas GFP is quenched under acidic conditions. Therefore, mCherry‐only (GFP‐negative) foci indicate mitophagy. It is noteworthy that mito-QC is connected to the cytoplasmic side of the OMM, and the GFP-mCherry fusion is vulnerable to proteasome assault. After CCCP treatment, OMM labeling largely disappears, coupled with the possible presence of fluorescence resonance energy transfer (FRET) between EGFP and mCherry, suggesting that FIS1 tag may be inappropriate for monitoring mitophagy.^363^ Although mt-Keima and Mito‐QC are currently popular mitophagy probes, they are difficult to be used for detecting mitophagy flux clinically due to their inevitable defects. Recently, a newly developed mitophagy probe, mito-SRAI, can detect mitophagy flux in fixed and living samples and has been used for high-throughput screening of therapeutic mitophagy inducers in vitro.^364^ Mito-SRAI is a TOLLES-YPet fluorescent protein targeting the mitochondrial matrix and is resistant to the lysosomal environment. TOLLES remains intact regardless of mitophagy activity, whereas YPet is efficiently degraded by mitophagy, allowing quantification of FRET measurements. Since mito-SRAI has well-characterized fluorescence and biochemical properties, its discovery will greatly accelerate the search on mitophagy modulators, and it may even bring great hope to the clinical detection of mitophagy flux.

Therapies targeting mitophagy are currently in ongoing or recently completed clinical trials aimed at improving mitophagy and ameliorating mitochondrial dysfunction. Urolithin A, a first-in-class natural compound, can induce mitophagy in vivo and in vitro. Urolithin A can activate mitophagy to improve neuroinflammation and delay aging.^365,366^ A clinical trial of urolithin A in immune health is currently recruiting patients (NCT05735886). Resveratrol is a natural polyphenol compound that exhibits antioxidant properties.^367^ It has been confirmed to play a role in multiple diseases via regulating mitophagy, and has been tested extensively in different clinical trials (NCT02123121, NCT04449198, NCT03728777, NCT02245932). More clinical studies are summarized in Table 3. Overall, clinical trials on direct modulation of mitophagy are lacking, and most mitophagy inducers in clinical trials show pleiotropic effects with unidentified mechanisms. Understanding how to specifically target mitochondria to regulate mitophagy and how to specifically detect mitophagy flux may bring breakthroughs for the application of mitophagy-inducing agent-targeted therapy in clinical trials.

**Table 3:** Clinical trials targeting mitophagy and mitochondrial function

Intervention/treatment	Disease	Phase	Status and results	Trial identifier
Urolithin A	Immune health	Not applicable	Recruiting	NCT05735886
Synthroid	Non-alcoholic fatty liver disease	Phase 2	Not yet recruiting	NCT05526144
Other: Strength training Protein supplementation	Skeletal muscle aging	Not applicable	Completed	NCT03326648
Mitopure	Skeletal muscle aging	Not applicable	Completed	NCT03283462
Resveratrol	Skeletal muscle diseases	Early phase 1	Recruiting	NCT04449198
Resveratrol	Skeletal muscle disease	Not applicable	Completed	NCT03728777
Resveratrol	Aging	Phase 2	Completed	NCT02123121
Resveratrol	Chronic obstructive pulmonary disease	Not applicable	Completed	NCT02245932
Salbutamol	Insulin resistance	Not applicable	Completed	NCT04558190
Metformin	Metabolic diseases	Phase 4	Completed	NCT01813929
Tetrahydrocannabinol Cannabidiol	Huntington’s disease	Phase 2	Completed	NCT01502046
Triheptanoin	Amyotrophic lateral sclerosis	Phase 1 Phase 2	Completed	NCT03506425
Lcatibant	Kidney disease	Phase 2	Completed	NCT03177798
Bevacizumab	Breast cancer	Early phase 1	Completed	NCT02806817

## Conclusions and perspectives

Since mitochondrial dysfunction underlies many diseases, mitophagy is currently a rapidly evolving field. In recent years, remarkable progress has been made in understanding of under what specific conditions and how mitophagy is activated on a cellular basis. The importance of mitophagy in a variety of diseases has been well documented. However, mitophagy is much more complex and will vary depending on metabolic state, stress condition and tissue developmental stage. Mitophagy mediated by multiple pathways exhibits parallelism, alternatives, and functional redundancy. Questions about the interplay between different mitophagy pathways, the spatio-temporal regulation rules of different mitophagy receptors under different physiological and pathological conditions, and the role of mitophagy components in vivo still exist and need to be resolved. One approach to consider is to study distinct organisms lacking single or multiple mitophagy receptors, stimulate them with diverse conditions, and cross them with genetic disease models to find out what specific pathways do under physiological or pathological conditions. Furthermore, with the recent development of in vivo models with various fluorescent reporter probes, it has become feasible to research mitophagy in living tissues. Using distinct models to study autophagy can provide different perspectives and reliable methods for studying the mechanism of mitophagy. Combining disease animal models with mitophagy imaging systems in vivo is beneficial to reveal the etiology and progression of diseases and facilitate translational research.

At present, there is still a gap in the research on the translating mitophagy mechanistic research into effective targeted drug. The classic common way to trigger mitophagy in vitro is to induce the depolarization of mitochondrial membrane potential by chemical reagents. Most of the mitophagy inducers still now are mitochondrial uncouplers or mitochondrial toxins, which have many limitations. The most important point is that the clinical efficacy of mitophagy modulators has not been fully established. With the development of new technology and artificial intelligence, the finding of new mitophagy regulators can be based on the discovery of natural products, rational drug design of specific target proteins, and phenotypic screening. A deep learning and better use of modern detection technology will be beneficial to the future research and verification of various mitophagy regulators. Although there are still many unanswered questions about mitophagy, we believe they can be addressed, and targeting mitophagy is a promising treatment for diseases for which there are presently no useful therapy.
